# Supplementary material for: Genipin prevents alpha-synuclein aggregation and toxicity by affecting endocytosis, metabolism and lipid storage
Source: Nat Commun. 2023 Apr 6;14:1918. doi: 10.1038/s41467-023-37561-2 (PMC10079842; doi:10.1038/s41467-023-37561-2)
Supplement: Supplementary file 1 — Supplementary Information [file 41467_2023_37561_MOESM1_ESM.pdf]

Supplementary Information

The small iridoid compound genipin prevents alpha-synuclein aggregation and toxicity by affecting endocytosis, metabolism and lipid storage

Rita Rosado-Ramos[1,2,3*], Gonçalo M. Poças[2*], Daniela Marques[3], Alexandre Foito[4], David M. Sevillano[5], Mafalda Lopes-da-Silva[3], Luís G. Gonçalves[2], Regina Menezes[1,3,6], Marcel Ottens[5], Derek Stewart[4], Alain Ibáñez de Opakua[7], Markus Zweckstetter[7,8], Miguel C. Seabra[3], César S. Mendes[3], Tiago Fleming Outeiro[7,9,10,11], Pedro M. Domingos[2], Cláudia Nunes dos Santos[1,2,3]

[1]iBET, Instituto de Biologia Experimental e Tecnológica, Oeiras, Portugal
[2]Instituto de Tecnologia Química e Biológica António Xavier, Universidade Nova de Lisboa (ITQB NOVA), Oeiras, Portugal
[3]iNOVA4Health, NOVA Medical School Faculdade de Ciências Médicas, NMS|FCM, Universidade Nova de Lisboa; Lisboa, Portugal.
[4]Environmental and Biochemical Sciences, The James Hutton Institute, DD2 5DA Dundee, Scotland
[5]Department of Biotechnology, Delft University of Technology, Delft, Netherlands
[6]CBIOS – Universidade Lusófona’s Research Center for Biosciences & Health Technologies, Campo Grande 376, 1749-024 Lisboa, Portugal
[7]German Center for Neurodegenerative Diseases (DZNE), 37075 Göttingen, Germany
[8]Max Planck Institute for Biophysical Chemistry, Department of Molecular Biology, Am Fassberg 11, 37077 Göttingen, Germany
[9]Department of Experimental Neurodegeneration, Center for Biostructural Imaging of Neurodegeneration, University Medical Center Göttingen, Göttingen, Germany
[10]Translational and Clinical Research Institute, Faculty of Medical Sciences, Newcastle University, NE2 4HH, United Kingdom
[11]Scientific employee with an honorary contract at German Center for Neurodegenerative Diseases (DZNE), 37075 Göttingen, Germany

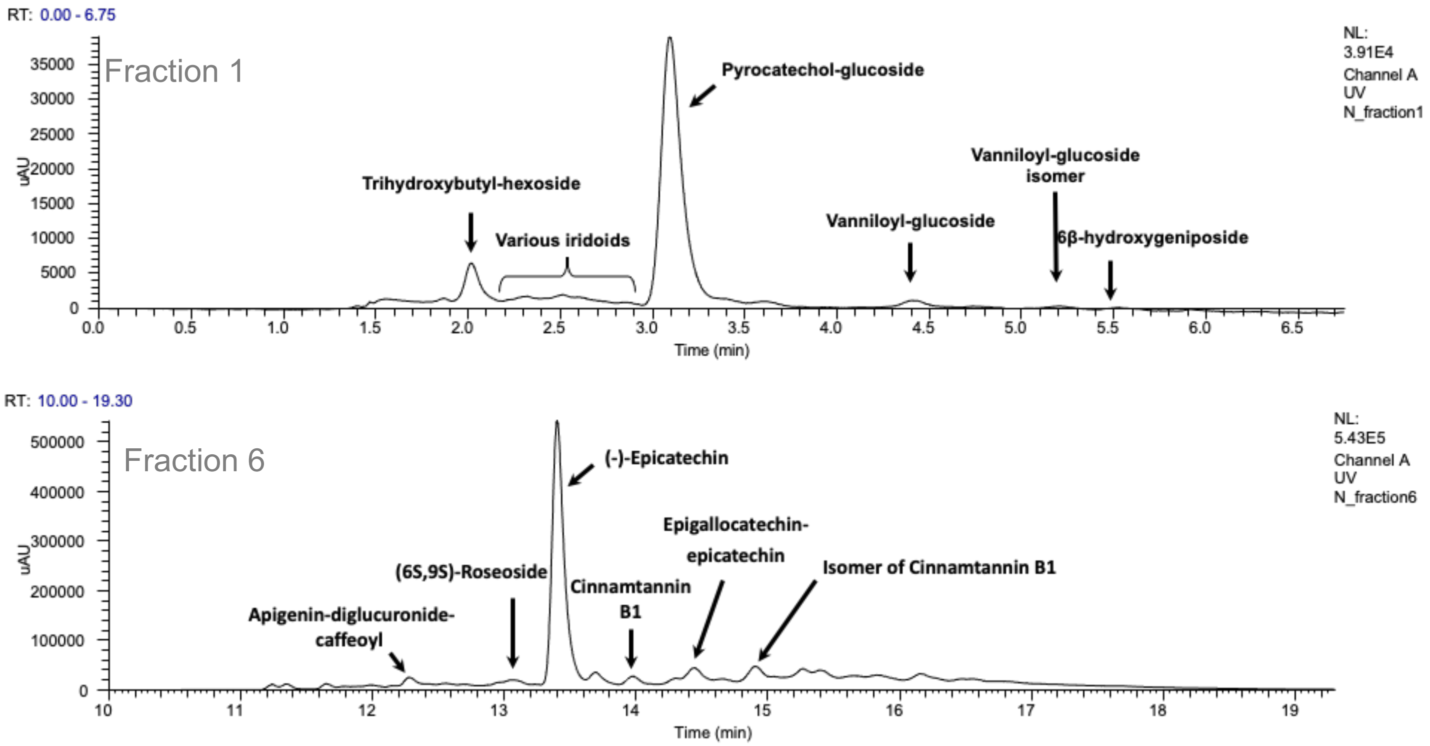

Supplementary Figure 1. Chromatographic profile of fractions 1 and 6 that retain bioactivity after the bioguided fractionation of *Corema album* L. leaf PEF extract. Fractions 1 and 6 were found to retain bioactivity (Figure 1A) and were analyzed by LC-PDA-MS (Thermo Orbitrap XL). The UV chromatograms of fractions 1 and 6 highlight the chemical diversity of both fractions. Annotations were done based on the mass spectral data collected from both positive and negative modes in full scan mode and data dependent MS2. Peaks present in these fractions were tentatively annotated by utilizing accurate mass (<3ppm), MS/MS fragmentation patterns and reference to retention time for previously analyzed standards (Supplementary table 1).

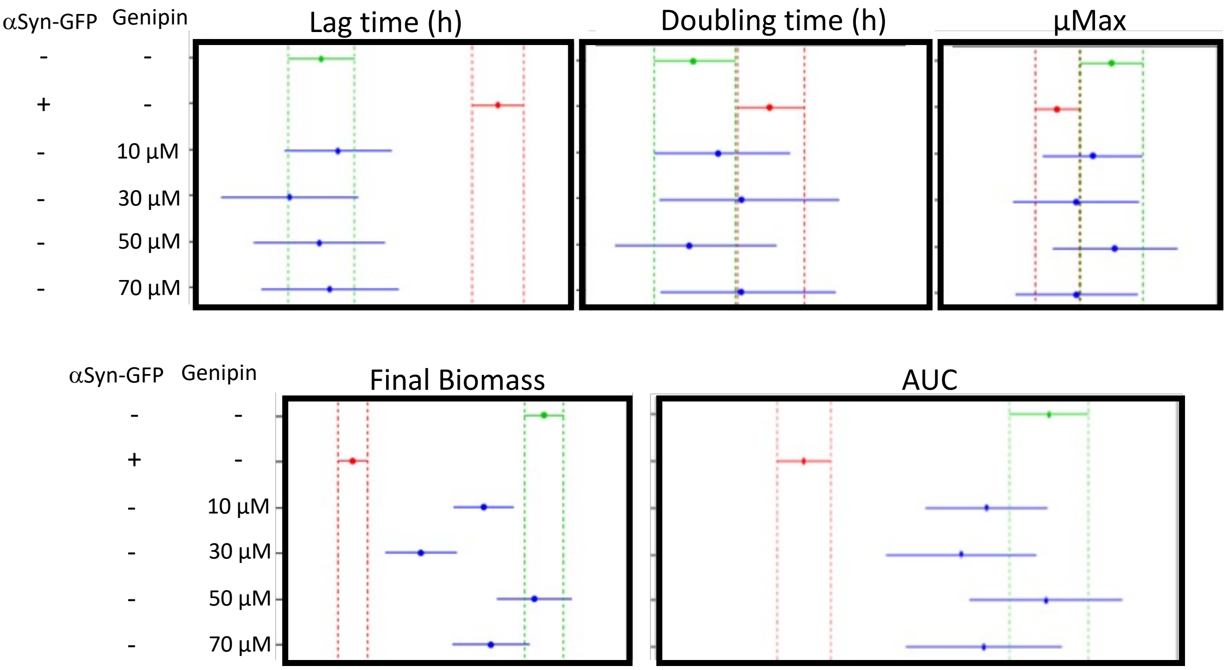

Supplementary Figure 2. Genipin toxicity characterized by growth curve parameters analysis. Control cells and cells expressing two copies of αSyn-GFP were pre-grown in raffinose medium until mid-log phase. The medium was discarded and cells were incubated in galactose medium. Control cells were supplemented or not with 10, 30, 50 or 70 µM of genipin for 24 h at 30ºC. OD_{600nm} was monitored every hour. Growth curves were treated using R package grofit to adjust a nonlinear parametric regression (model-based) and the growth parameters were estimated from the best model fit and the 95% confidence intervals were calculated.

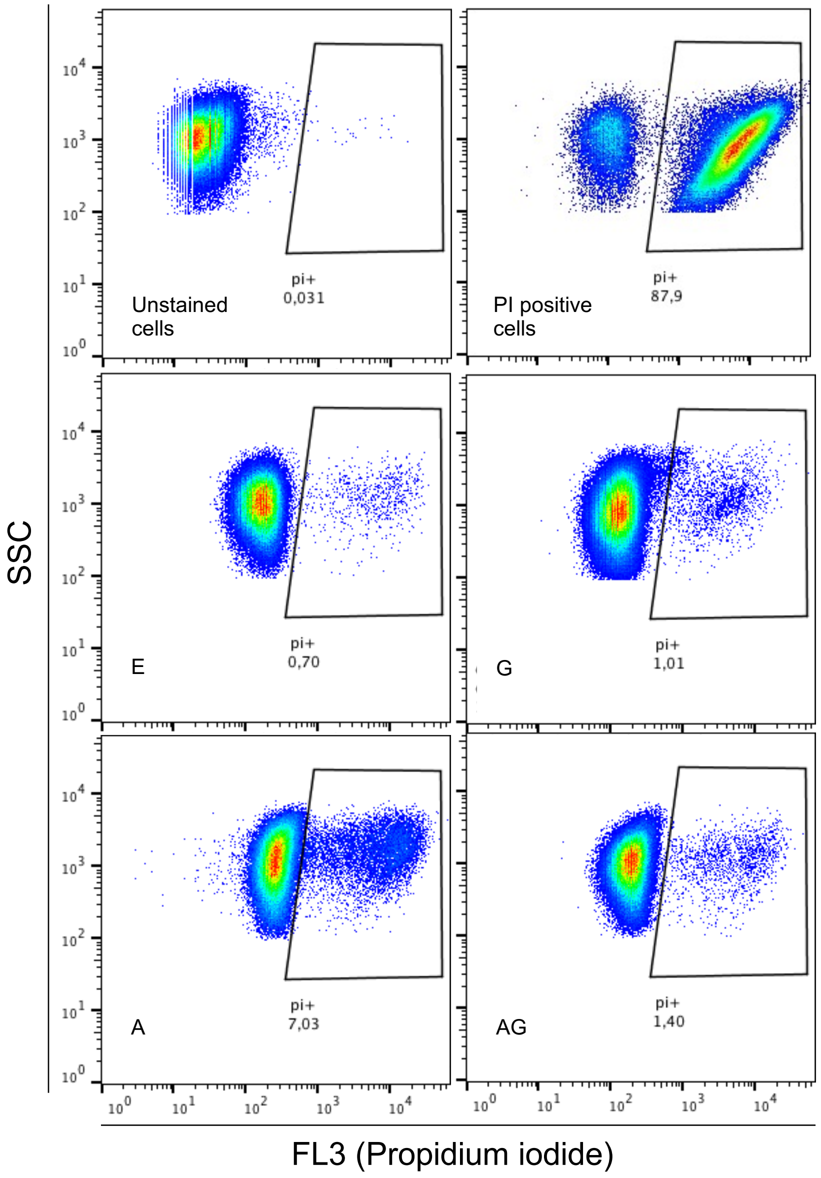

Supplementary Figure 3. Illustration of gating strategy of FCM data. Data analysis was performed using FlowJo software and a minimum of 100,000 events were collected for each experiment represented using Side Scatter (SSC) versus FL3 (Propidium Iodide). The represented gating strategy was based on both negative and positive controls for Propidium Iodide and it was used for all the dyes used, namely Dihydroethidium (DHE) and Neutral Lipids Sysmex Kit.

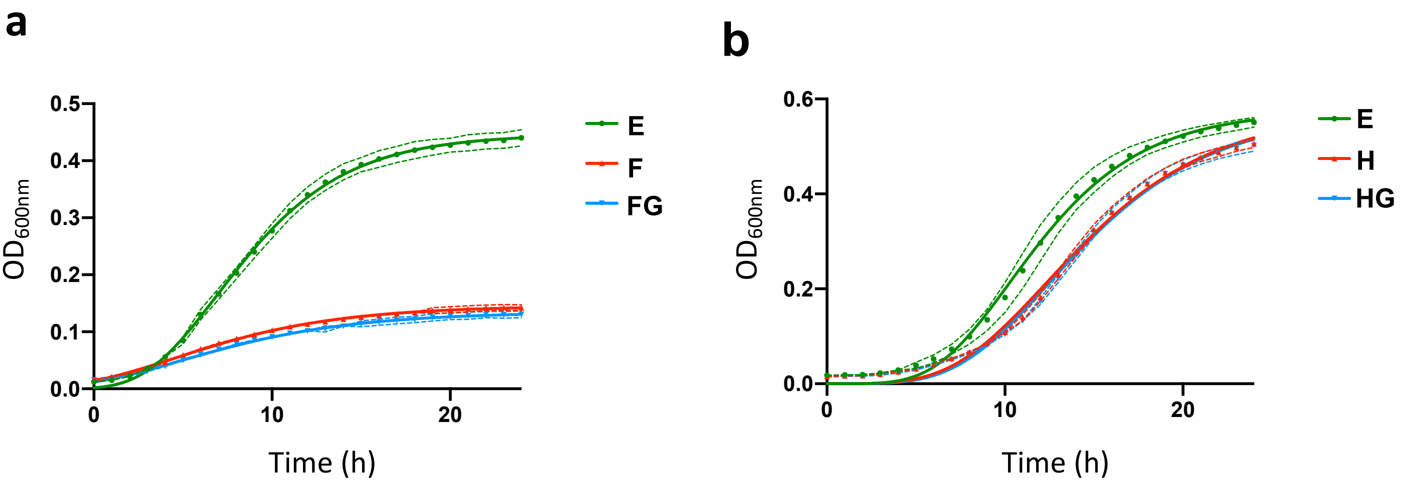

Supplementary Figure 4. Genipin bioactivity specificity for αSyn. Control cells and cells expressing Fused in sarcoma protein (FUS) (a) or huntingtin exon 1 (Htt) (b) were pre-grown in raffinose medium until mid-log phase. The medium was discarded and cells were incubated in galactose medium supplemented or not with 10 μM of genipin for 24 h at 30ºC. OD_{600nm} was monitored every hour. n=3. Control cells with empty vector – E, 2xFUS cells – F, 2xFUS cells + 10 μM genipin – FG , Htt exon 1-GFP cells – H, Htt exon 1-GFP cells + 10 μM genipin – HG.

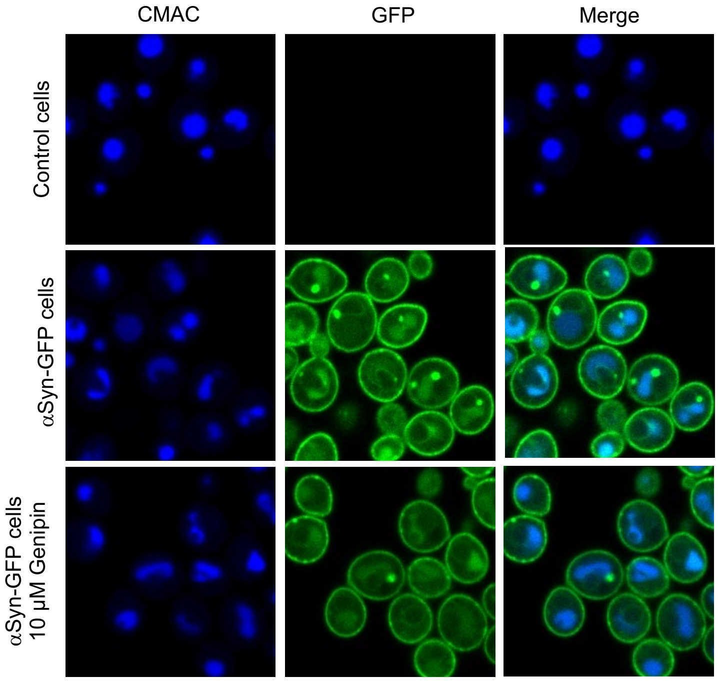

Supplementary Figure 5. Endocytosis independent blue-fluorescent chloromethyl derivative of aminocoumarin dye CMAC blue (CMAC) enters yeast cells and accumulate in the vacuole. Control cells and cells expressing two copies of αSyn-GFP were pre-grown in raffinose medium until mid-log phase. The medium was discarded and cells were incubated in galactose medium supplemented or not with 10 μM of genipin for 6 h at 30ºC. CMAC blue staining was performed according manufactures' instructions and visualized by confocal microscopy.

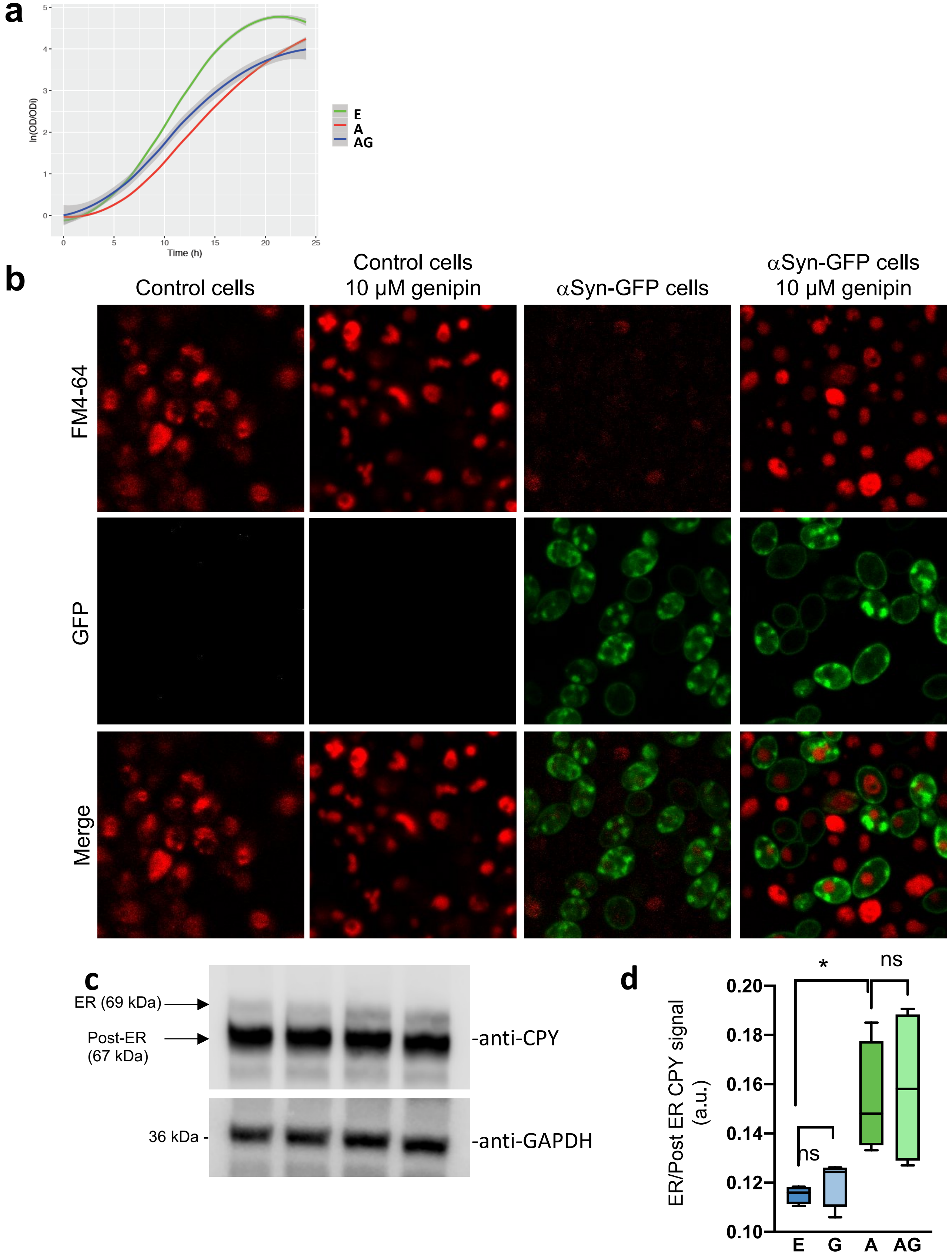

Supplementary Figure 6. Effect of genipin treatment in cellular trafficking of BY4741 WT strain. (a) Growth curves of αSyn-GFP yeast in the presence or not of genipin. OD_{600nm} was monitored every hour. Growth curves were treated using R package grofit to adjust a nonlinear parametric regression (model-based) and the growth parameters were estimated from the best model fit. 95 % confidence intervals were used to assess statistical differences between conditions. (b) Endocytosis evaluation by FM4-64 incubation by confocal microscopy. (c) ER accumulation of CPY assessed by western blot. (d) ER/Post ER ratio CPY was assessed by densitometry. (n=4, E vs A, *p=0.0228). Control cells with empty vector – E, Control cells + 10 μM genipin – G, αSyn-GFP cells – A, αSyn-GFP cells + 10 μM genipin – AG.

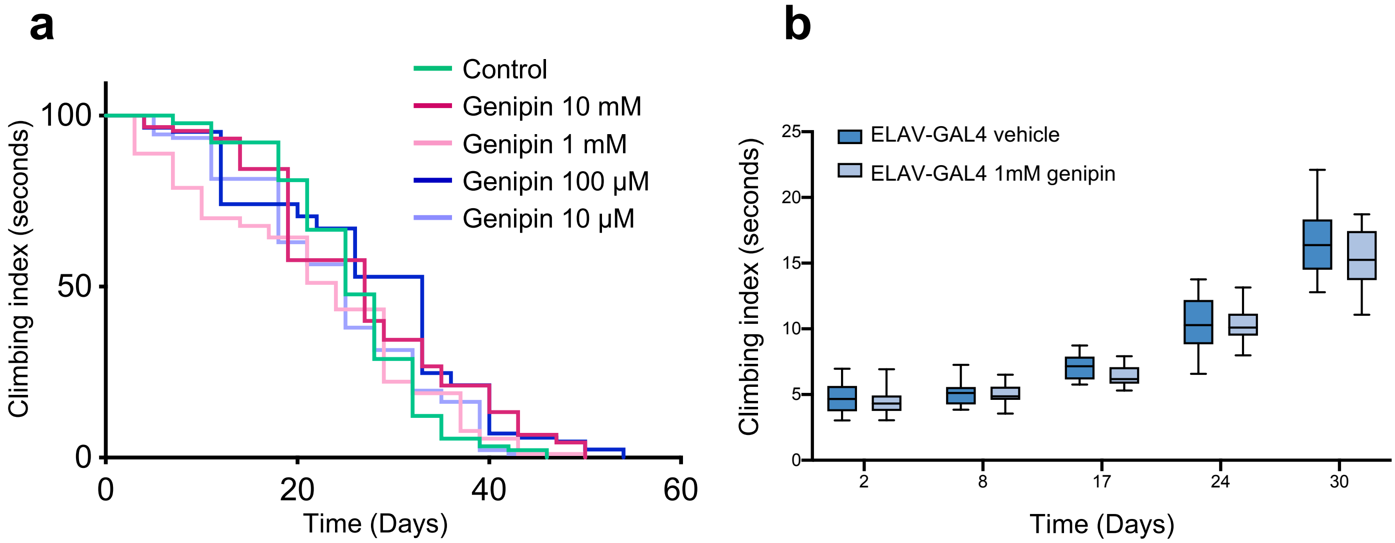

Supplementary Figure 7. Genipin presents no toxicity or effects in control flies. **(a)** Lifespan assessment of WT flies fed or not with genipin. Values on the Y-axis represent the percentage of flies alive at each time point analyzed. **(b)** Flies expressing control vector ELAV-GAL4 fed or not with 1 mM genipin. Climbing index represents the time (in seconds), it took for five males to climb 15 cm (mean± SEM).

Supplementary table 1a. List of annotated peaks found in the bioactive *C. album* L. leaf extract fractions 1. Fractions were analyzed on a Thermo Orbitrap XL LC-MS in positive and negative modes in full scan mode and data dependent MS2. Peaks present in these fractions were tentatively annotated by utilizing accurate mass (<1ppm), MS/MS fragmentation patterns and reference to retention time for previously analyzed standards. Tentatively identified iridoids are highlighted in blue.

RT[a]	m/z		Molecular formula	Tentative id	ID Level[b]
	Pos	Neg			
2.13	-	191.0198[M-H]$^-$	$C_6H_8O_7$	Likely organic acid	4
2.09	-	283.1031[M-H]$^-$	$C_{10}H_{20}O_9$	Trihydroxybutyl-hexoside	3
2.19	-	253.0929 [M-H]$^-$ / [M+FA]$^-$	$C_9H_{18}O_8$ / $C_8H_{16}O_6$	Dihydroxupropanoyl-glucoside / Ethyl-glucoside	3
2.15	-	295.1032 [M-H]$^-$ / [M+FA]$^-$	$C_{11}H_{20}O_9$ / $C_{10}H_{18}O_7$	Methyl xylopyranosyl-xylopyranoside / Potential PEG derivative	3/4
2.21	-	421.1348 [M-H]$^-$	$C_{17}H_{26}O_{12}$	Potential iridoid	4
2.39	-	611.1442 [M-H]$^-$	$C_{36}H_{24}N_2O_8$	5,5'-[1,4-Phenylenebis(oxy)]bis[2-(4-methoxyphenyl)-1H-isoindole-1,3(2H)-dione] / 4,8-Bis(4-methoxyphenoxy)-2,6-diphenylpyrrolo[3,4-f]isoindole-1,3,5,7(2H,6H)-tetrone	3
2.49	-	451.1457 [M+FA]$^-$	$C_{17}H_{26}O_{11}$	Potential iridoid	3
2.56	289.0917 [M+H]$^+$	333.0825 [M+FA]$^-$	$C_{12}H_{16}O_8$	Pyrogalloyl-glucoside	2
2.76	-	265.0928 [M-H]$^-$	$C_{10}H_{18}O_8$	Unknown	4
2.94	-	451.1456	$C_{17}H_{26}O_{11}$	Potential isomer of 2.49	3
2.91	-	441.1165 [M-H]$^-$	$C_{18}H_{22}N_2O_{11}$	1-(2,3,4,6-Tetra-O-acetylhexopyranosyl)-2,4(1H,3H)-pyrimidinedione	3
3.14 (3.11)	290.1233 [M+NH$_4$]$^+$	317.0872 [M+FA]$^-$	$C_{12}H_{16}O_7$	Pyrocatechol-glucoside	2
3.43	-	341.1085 [M+FA]$^-$	$C_{11}H_{20}O_9$	Dimethyl (6S)-3-deoxy-6-[(1R,2R)-1,2,3-trihydroxypropyl]-α-L-threo-hex-2-ulopyranosidonate	3
3.48	-	685.1824 [M-H]$^-$	$C_{26}H_{38}O_{21}$	Unknown	4
3.54	-	645.1874 [M-H]$^-$	$C_{24}H_{38}O_{20}$	4-Deoxy-α-L-threo-hex-4-enopyranuronosyl-(1->4)-β-D-glucopyranosyl-(1->4)-6-deoxy-α-L-mannopyranosyl-(1->3)-β-D-glucopyranose	3
3.48	-	297.1190 [M+FA]$^-$	$C_{10}H_{20}O_7$	4-Hydroxybutyl β-D-glucopyranoside	3
3.43	-	553.1768 [M-H]$^-$	$C_{35}H_{26}N_2O_5$	2'-(1,3-Benzodioxol-5-ylcarbonyl)-1'-benzoyl-5'-methyl-1',2'-dihydro-3a'H-spiro[indole-3,3'-pyrrolo[1,2-a]quinolin]-2(1H)-one	3
3.64	-	421.1346 [M+FA]$^-$	$C_{16}H_{24}O_{10}$	Loganate (an iridoid)	3
3.79	-	265.0926 [M-H]$^-$	$C_{10}H_{18}O_8$	Ethyl glucopyranosyloxy acetate	3

3.90	-	297.1188 $[M+FA]^-$	$C_{10}H_{20}O_7$	Potential isomer of 4-Hydroxybutyl β-D-glucopyranoside	3
3.90	-	777.2296 $[M-H]^-$	$C_{29}H_{46}O_{24}$	Unknown	4
4.05	-	309.1187 $[M-H]^-$ / $[M+FA]^-$	$C_{12}H_{22}O_9$ / $C_{11}H_{20}O_7$	1,6-Dideoxy-β-D-fructofuranosyl α-D-glucopyranoside / (2E)-4-Hydroxy-3-methyl-2-buten-1-yl β-D-glucopyranoside	3
4.05	-	647.2030 $[M-H]^-$	$C_{24}H_{40}O_{20}$	Unknown	4
4.31	-	331.0667 $[M-H]^-$	$C_{13}H_{16}O_{10}$	Gallic acid-hexoside	2
4.47	-	329.0874 $[M-H]^-$	$C_{14}H_{18}O_9$	Vanniloyl-glucoside	2
4.55	-	309.1188 $[M-H]^-$	$C_{12}H_{22}O_9$ / $C_{11}H_{20}O_7$	Potential isomer from 4.05	3
5.27	-	329.0873 $[M-H]^-$	$C_{14}H_{18}O_9$	Potential isomer of Vanniloyl-glucoside	2
5.27	-	779.2448 $[M-H]^-$	$C_{24}H_{48}N_2O_{26}$	Unknown	4
5.60	-	449.1296 $[M+FA]^-$	$C_{17}H_{24}O_{11}$	6β-hydroxygeniposide	3
5.75	-	645.1874 $[M-H]^-$	$C_{24}H_{38}O_{20}$	Potential isomer of peak at 3.40	3
5.90	314.1446 $[M+H]^+$	295.1031 $[M-H]^-$	$C_{11}H_{20}O_9$	1,5-Anhydro-2-O-β-L-arabinopyranosyl-D-mannitol	2
5.93	-	385.0983 $[M-H]^-$	$C_{26}H_{14}N_2O_2$	8,16-Dihydrobenzo[mn]phenanthridino[6,7-bc]acridine-1,9-dione	3
6.86	-	345.0824 $[M-H]^-$	$C_{14}H_{18}O_{10}$	2-Acetyl-3,5,6-trihydroxyphenyl β-D-glucopyranoside	3
2.07	332.1340 $[M+H]^+$	-	$C_{14}H_{21}NO_8$	1,3-Dioxooctahydro-1H-isoindol-4-yl β-D-glucopyranoside	3
2.07	202.1800 $[M+H]^+$	-	$C_{11}H_{23}NO_2$	Unknown	4
2.16	284.1703 $[M+H]^+$	-	$C_{11}H_{25}NO_7$	Unknown	4
2.41	307.0831 $[M+H]^+$	-	$C_{18}H_{12}NO_4$	Unknown	4
2.46	227.0913 $[M+H]^+$	-	$C_{11}H_{14}O_5$	Genipin (potential in-source fragment of an iridoid derivate)	3
2.46	424.1813 $[M+H]^+$	-	$C_{17}H_{29}NO_{11}$	Potential iridoid derivative of Genipin	4
2.46	452.2125 $[M+H]^+$	-	$C_{19}H_{33}NO_{11}$	Derivative of 424 compouind (C2H4)	4
2.70	117.0544 $[M+H]^+$	-	$C_5H_8O_3$	1-Acetoxyacetone	3
2.75	284.1339 $[M+NH_4]^+$	-	$C_{10}H_{18}O_8$	Hydroxybutyl-glucoside (isomer of peak at 3.45)	2
2.79	312.1652 $[M+NH_4]^+$	-	$C_{12}H_{22}O_8$	Bis-PEG4-acid / 6-O-(2-Hydroxyhexanoyl)-D-glucopyranose	2
2.89	227.0913 $[M+H]^+$	-	$C_{11}H_{14}O_5$	Isomer of Genipin derivative (potential in-source fragment of an iridoid derivate)	3
3.38	342.1759 $[M+H]^+$	-	$C_{13}H_{24}O_9$	Methyl 3-(β-D-glucopyranosyloxy)-5-hydroxyhexanoate	3
3.43	268.1039 $[M+H]^+$	-	$C_9H_{17}NO_8$	Neuramininc acid	3

3.48	298.1859 [M+H]$^+$	-	$C_{12}H_{27}NO_7$	2-Amino-3-[(2-methoxyethoxy)methoxy]-2-{[(2-methoxyethoxy)methoxy]methyl}-1-propanol	3
3.79	312.1652 [M+H]$^+$	-	$C_{12}H_{22}O_8$	Bis-PEG4-acid isomer / 6-O-(2-Hydroxyhexanoyl)-D-glucopyranose isomer	2
3.94	298.1859 [M+H]$^+$	-	$C_{12}H_{27}NO_7$	Isomer of 3.48 peak	3
4.44	348.1290 [M+H]$^+$	-	$C_{14}H_{21}NO_9$	(2E)-[(2S,3S,4S,6S)-6-(β-D-Glucopyranosyloxy)-2,3,4-trihydroxycyclohexylidene]acetonitrile	3
4.54	310.1860 [M+NH$_4$]$^+$	-	$C_{13}H_{24}O_7$	1-O-heptanoyl- β-D-glucopyranoside	3
5.19	310.1860 [M+NH$_4$]$^+$	-	$C_{13}H_{24}O_7$	Isomer of heptanoyl-glucopyranoside	3
2.13	-	191.0198[M-H]$^-$	$C_6H_8O_7$	Likely organic acid	4
2.09	-	283.1031[M-H]$^-$	$C_{10}H_{20}O_9$	Trihydroxybutyl-hexoside	3
2.19	-	253.0929 [M-H]$^-$ / [M+FA]$^-$	$C_9H_{18}O_8$ / $C_8H_{16}O_6$	Dihydroxupropanoyl-glucoside / Ethyl-glucoside	3
2.15	-	295.1032 [M-H]$^-$ / [M+FA]$^-$	$C_{11}H_{20}O_9$ / $C_{10}H_{18}O_7$	Methyl xylopyranosyl-xylopyranoside / Potential PEG derivative	3/4
2.21	-	421.1348 [M-H]$^-$	$C_{17}H_{26}O_{12}$	Potential iridoid	4
2.39	-	611.1442 [M-H]$^-$	$C_{36}H_{24}N_2O_8$	5,5'-[1,4-Phenylenebis(oxy)]bis[2-(4-methoxyphenyl)-1H-isoindole-1,3(2H)-dione] / 4,8-Bis(4-methoxyphenoxy)-2,6-diphenylpyrrolo[3,4-f]isoindole-1,3,5,7(2H,6H)-tetrone	3
2.49	-	451.1457 [M+FA]$^-$	$C_{17}H_{26}O_{11}$	Potential iridoid	3
2.56	289.0917 [M+H]$^+$	333.0825 [M+FA]$^-$	$C_{12}H_{16}O_8$	Pyrogalloyl-glucoside	2
2.76	-	265.0928 [M-H]$^-$	$C_{10}H_{18}O_8$	Unknown	4
2.94	-	451.1456	$C_{17}H_{26}O_{11}$	Potential isomer of 2.49	3
2.91	-	441.1165 [M-H]$^-$	$C_{18}H_{22}N_2O_{11}$	1-(2,3,4,6-Tetra-O-acetylhexopyranosyl)-2,4(1H,3H)-pyrimidinedione	3
3.14 (3.11)	290.1233 [M+NH$_4$]$^+$	317.0872 [M+FA]$^-$	$C_{12}H_{16}O_7$	Pyrocatechol-glucoside	2
3.43	-	341.1085 [M+FA]$^-$	$C_{11}H_{20}O_9$	Dimethyl (6S)-3-deoxy-6-[(1R,2R)-1,2,3-trihydroxypropyl]-α-L-threo-hex-2-ulopyranosidonate	3
3.48	-	685.1824 [M-H]$^-$	$C_{26}H_{38}O_{21}$	Unknown	4
3.54	-	645.1874 [M-H]$^-$	$C_{24}H_{38}O_{20}$	4-Deoxy-α-L-threo-hex-4-enopyranuronosyl-(1->4)-β-D-glucopyranosyl-(1->4)-6-deoxy-α-L-mannopyranosyl-(1->3)-β-D-glucopyranose	3
3.48	-	297.1190 [M+FA]$^-$	$C_{10}H_{20}O_7$	4-Hydroxybutyl β-D-glucopyranoside	3

[a]RT – Retention time; [b]ID Level - The Metabolomics Standards Initiative defined four levels of metabolite identification confidence to which the manuscript has adhered to. Confidently identified compounds are classified as a level 1and require evidence based on two or more orthogonal properties with an authentic chemical standard analysed under identical analytical conditions. Putatively annotated compounds are classified as level 2, and are based upon physicochemical properties and/or spectral similarity with public commercial spectral libraries, without reference to authentic chemical standards. Putatively annotated compound classes are categorized as level 3, and are based upon characteristic physicochemical properties of a chemical class of compounds, or by spectral similarity to know compounds of a chemical class. Unknown compounds are classified as level 4 and although they remain unidentified and unclassified, these metabolites can still be differentiated and quantified based upon spectral data[8].

Supplementary table 1b. List of annotated peaks found in the bioactive *C. album* L. leaf extract fractions 6. Fractions were analyzed on a Thermo Orbitrap XL LC-MS in positive and negative modes in full scan mode and data dependent MS2. Peaks present in these fractions were tentatively annotated by utilizing accurate mass (<1ppm), MS/MS fragmentation patterns and reference to retention time for previously analyzed standards. Tentatively identified iridoids are highlighted in blue.

RT[a]	m/z		Molecular formula	Tentative id	ID Level[b]
	Pos	Neg			
10.07	-	513.1606 [M-H]$^-$	$C_{23}H_{30}O_{13}$	(1S,4aR,5S,7aS)-7-(Hydroxymethyl)-1-[(2,3,4-tri-O-acetyl-β-D-glucopyranosyl)oxy]-1,4a,5,7a-tetrahydrocyclopenta[c]pyran-5-yl acetate (potential iridoid)	3
10.62	-	497.1657 [M-H]$^-$	$C_{23}H_{30}O_2$	Nishindaside (potential iridoid)	3
10.86	-	767.1450 [M-H]$^-$	-	Unknown	4
10.72	-	625.1035 [M-H]$^-$	$C_{26}H_{26}O_{18}$	Ellagic acid-glucoside-xyloside	2
10.94	-	335.1131 [M-H]$^-$	$C_{17}H_{20}O_7$	Methyl (3R,4S,5R)-3,5-dihydroxy-4-{[3-(4-hydroxyphenyl)propanoyl]oxy}-1-cyclohexene-1-carboxylate	3
11.15	-	785.1556 [M-H]$^-$	$C_{36}H_{34}O_{20}$	Unknown	4
11.15	-	879.1760 [M-H]$^-$	$C_{45}H_{36}O_{19}$	Unknown	4
11.73	473.1289 [M+H]$^+$	-	$C_{20}H_{24}O_{13}$	7-Hydroxy-1-benzofuran-2-carboxylic acid- Hexoside-Pentoside	3
11.65	643.1508 [M+H]$^+$	-	$C_{27}H_{30}O_{18}$	Myricetin di-glucoside	2
11.83	643.1507 [M+H]$^+$	-	$C_{27}H_{30}O_{18}$	Myricetin di-glucoside isomer	2
11.63	195.0651 [M+H]$^+$	-	$C_{10}H_{10}O_4$	Ferulic acid	1/2
11.47	503.1871 [M+H]$^+$	-	-	Unknown	4
11.32	655.1143 [M+H]$^+$	-	$C_{27}H_{26}O_{19}$	Myricetin Hexoside-citrate	3
11.63	374.1447 [M+H]$^+$	-	$C_{16}H_{23}NO_9$	2-Amino-3-(4-hydroxy-3-{[(2R,3R,4S,5S,6R)-3,4,5-	3

				trihydroxy-6-(hydroxymethyl)tetrahydro-2H-pyran-2-yl]oxy}phenyl)-2-methylpropanoic acid	
11.93	341.0867 [M+H]$^+$	-	$C_{15}H_{16}O_9$	Esculetin 6-O-glucoside	3
12.03	213.1119 [M+H]$^+$ 392.1913 [M+H]$^+$ 420.2223 [M+H]$^+$	-	$C_{11}H_{16}O_4$ $C_{17}H_{29}NO_9$ $C_{19}H_{33}NO_9$	Trimethoxyphenyl ethanol (2S)-2-Hydroxy-2-[(2R,5R,6R)-2-methoxy-5,6-dimethyl-4-methylenetetrahydro-2H-pyran-2-yl]-N-[(2S,3S,4R,5R,6S)-3,4,5-trihydroxy-6-(hydroxymethyl)tetrahydro-2H-pyran-2-yl]acetamide<br><br>Unknown	3
12.11	372.2592 [M+2Na-H]$^+$	-	$C_{20}H_{39}O_3$	Unknown	3
12.30	257.1382 [M+H]$^+$	-	$C_{13}H_{20}O_5$	2-[4-(3-Hydroxypropyl)-2-methoxyphenoxy]-1,3-propanediol	2
12.40	769.1611 [M+H]$^+$	-	$C_{36}H_{32}O_{19}$	Apigenin-diglucuronide-caffeoyl	3
12.59	420.1863 [M+H]$^+$ / [M+NH$_4$]$^+$	-	$C_{18}H_{29}NO_{10}$ $C_{18}H_{26}O_{10}$	Unknown Benzyl 6-O-β-D-xylopyranosyl-β-D-glucopyranoside	3
12.59	448.2174 [M+H]$^+$ / [M+2Na-H]$^+$	-	$C_{20}H_{33}NO_{10}$	(2S)-2-Hydroxy-N-[(4R,4aS,6R,7R,8S)-7-hydroxy-6-(hydroxymethyl)-8-methoxyhexahydropyrano[3,2-d][1,3]dioxin-4-yl]-2-[(2R,5R,6R)-2-methoxy-5,6-dimethyl-4-methylenetetrahydro-2H-pyran-2-yl]acetamide (non - preferred name)	4
12.55	769.1611 [M+H]$^+$	-	$C_{36}H_{32}O_{19}$	Isomer of Apigenin-diglucuronide-caffeoyl	3
12.79	464.2125 [M+H]$^+$	-	$C_{20}H_{33}NO_{11}$	N-[(2R,3R,4R,5S,6R)-4,5-Dihydroxy-6-(hydroxymethyl)-2-{[(3aR,5R,5aS,8aS,8bR)-2,2,7,7-tetramethyltetrahydro-3aH-bis[1,3]dioxolo[4,5-b:4',5'-d]pyran-5-yl]methoxy}tetrahydro-2H-pyran-3-yl]acetamide	3
12.87	416.2855 [M+2Na-H]$^+$ 388.2543 [M+NH$_4$]$^+$	-	$C_{16}H_{34}O_9$	Octanethyl glycol (plasticizer)	2
13.09	387.2019 [M+H]$^+$	-	$C_{19}H_{30}O_8$	(6S,9S)-Roseoside	2
13.36	291.0864 [M+H]$^+$ 581.1656 [2M+H]$^+$	-	$C_{15}H_{14}O_6$	(-)-Epicatechin	1

13.54	460.3112 [M+2Na-H]+ 432.2802 [M+NH4]+ 415.2537 [M+H]+	-	$C_{18}H_{38}O_{10}$	Octaoxahexacosane (PEG derivative)	2
13.82	462.2331 [M+2Na-H]+ 434.2020 [M+NH4]+	-	$C_{19}H_{28}O_{10}$	2-Phenylethyl 6-O-β-D-xylopyranosyl-β-D-glucopyranoside	2
13.92	865.1976 [M+H]+	-	$C_{45}H_{36}O_{18}$	Cinnamtannin B1 (trimer)	2
14.16	459.2797 [M+H]+ 476.3064 [M+NH4]+ 504.3376 [M+2Na-H]+	-	$C_{20}H_{42}O_{11}$	Decaethylene glycol (PEG derivative)	2
13.63	853.1978 [M+H]+	-	$C_{44}H_{36}O_{18}$	Potential tannin (trimer)	4
13.73	593.1291 [M+H]+	-	$C_{30}H_{24}O_{13}$	Epigallocatechin-epicatechin (dimer)	2
14.42	1153.2609 [M+H]+	-	$C_{60}H_{48}O_{24}$	Cinnamtannin B2 (tetramer)	2
14.42	593.1289 [M+H]+	-	$C_{30}H_{24}O_{13}$	Isomer of Epigallocatechin-epicatechin (dimer)	2
14.61	1441.3241 [M+H]+	-	$C_{75}H_{60}O_{30}$	Polymer derived from Cinnamtannin-type compound (tetramer)	3
14.66	849.2025 [M+H]+	-	$C_{45}H_{36}O_{17}$	Similar to Cinnamtannin B1 (one less oxygen) (trimer)	3
14.86	865.1976 [M+H]+	-	$C_{45}H_{36}O_{18}$	Isomer of Cinnamtannin B1 (trimer)	2
15.26	577.1341 [M+H]+	-	$C_{30}H_{24}O_{12}$	Epicatechin-Epicatechin (dimer)	2
15.55	577.1341 [M+H]+	-	$C_{30}H_{24}O_{12}$	Isomer of Epicatechin-Epicatechin (dimer)	2
15.86	720.1582	-	-	Unknown	4
15.65	1151.2446 [M+H]+	-	$C_{60}H_{46}O_{24}$	Monounsaturated form of Cinnamtannin B2 (tetramer)	3
16.15	720.1582 [M+2H]2+	-	$C_{75}H_{58}O_{30}$	Monounsaturated form of Cinnamtannin-type (pentamer)	3
16.54	864.1891 [M+2H]2+	-	$C_{90}H_{70}O_{36}$	Cinnamtannin-type compound (hexamer)	3

[a]RT – Retention time; [b]ID Level - The Metabolomics Standards Initiative defined four levels of metabolite identification confidence to which the manuscript has adhered to. Confidently identified compounds are classified as a level 1and require evidence based on two or more orthogonal properties with an authentic chemical standard analysed under identical analytical conditions. Putatively annotated compounds are classified as level 2, and are based upon physicochemical properties and/or spectral similarity with public commercial spectral libraries, without reference to authentic chemical standards. Putatively annotated compound classes are categorized as level 3, and are based upon characteristic physicochemical properties of a chemical class of compounds, or by spectral similarity to know compounds of a chemical class. Unknown compounds are classified as level 4 and although they remain unidentified and

unclassified, these metabolites can still be differentiated and quantified based upon spectral data[8].

Supplementary table 2. Effect of αSyn-GFP overexpression on yeast transcriptome. 63 genes differentially expressed (FDR<0.05) in cells overexpressing αSyn-GFP incubated with 10 µM of genipin comparing with cells carrying the empty vector.

ORF	ID	logFC	logCPM	PValue	FDR
YFL068W	YFL068W	-1.5031877	2.42741067	1.06E-05	2.00E-03
YIL165C	YIL165C	-1.3521125	5.82885977	2.15E-05	2.00E-03
YFL067W	YFL067W	-1.3457829	1.74017426	0.00123961	3.50E-02
YBL108C-A	PAU9	-1.3117746	1.994861	0.00082138	2.66E-02
YAL061W	BDH2	-1.2561854	7.97884125	0.00051015	1.93E-02
YDL235C	YPD1	-1.23162	6.50521637	5.18E-05	3.50E-03
YNL322C	KRE1	-1.2058142	7.34264712	8.96E-05	5.00E-03
RME3	RME3	-1.1760336	8.49493081	0.00079142	2.64E-02
YPL269W	KAR9	-1.1729818	8.48156272	0.00058885	2.09E-02
YHL034C	SBP1	-1.160961	7.17159379	3.24E-05	2.58E-03
YGL249W	ZIP2	-1.1187061	8.63843968	0.00076595	2.62E-02
YAR023C	YAR023C	-1.0473373	3.85226441	0.00022092	9.65E-03
YLL054C	YLL054C	-1.0449635	8.83480873	0.00120442	3.48E-02
YCL052C	PBN1	-0.967683	8.36256734	0.00128354	3.58E-02
YFL050C	ALR2	-0.9280723	9.57667146	0.00013554	7.04E-03
YKL117W	SBA1	-0.8591759	3.95715988	0.00081085	2.66E-02
YJL212C	OPT1	-0.8525683	9.93579755	7.12E-05	4.40E-03
YEL017C-A	PMP2	-0.84406	3.7404141	0.00055046	1.98E-02
YOL154W	ZPS1	-0.8281397	7.77047649	0.00086255	2.75E-02
YDL234C	GYP7	-0.8161198	10.0031478	7.30E-05	4.40E-03
YEL062W	NPR2	-0.8054442	9.77351633	9.48E-05	5.16E-03
YKL212W	SAC1	-0.7790162	9.88764411	0.0001073	5.70E-03
YAL060W	BDH1	-0.7471766	9.53094248	0.00018282	8.83E-03
YHL033C	RPL8A	-0.7410919	8.92485635	0.00015456	7.67E-03
YLL052C	AQY2	-0.7387157	8.31377405	0.00034378	1.37E-02
YLL053C	YLL053C	-0.7278973	8.31500789	0.00018595	8.83E-03
YDL134C	PPH21	-0.7248458	4.55778799	0.00102377	3.13E-02
YML117W	NAB6	-0.7236058	10.0819914	0.00036355	1.42E-02
YGL248W	PDE1	-0.7205834	9.11558113	0.00022912	9.65E-03
YNL321W	VNX1	-0.6782443	9.91710771	0.00053829	1.97E-02
YPL268W	PLC1	-0.6325021	9.72338212	0.0012051	3.48E-02
YEL017W	GTT3	-0.5987355	6.73550981	0.00153605	4.18E-02
YAR008W	SEN34	0.62358577	5.50941357	0.00186083	4.94E-02
YKL170W	MRPL38	0.62774499	7.44748656	0.0006917	2.41E-02
YKL207W	EMC3	0.63216102	5.9657022	0.00176845	4.76E-02
YPL232W	SSO1	0.63891451	7.4153447	0.00099864	3.10E-02
YBL033C	RIB1	0.66008967	5.43475397	0.00094736	2.98E-02
YJL206C	YJL206C	0.66365771	8.42873278	0.00053343	1.97E-02
YNL282W	POP3	0.68102126	6.64824487	0.0003752	1.44E-02
YAL059W	ECM1	0.7122457	5.36006543	0.00077619	2.62E-02
YKL181W	PRS1	0.71380843	7.77008717	0.00022842	9.65E-03
YML111W	BUL2	0.75021645	9.18155012	8.33E-05	4.77E-03
YIL158W	AIM20	0.77058015	7.06572788	0.00015201	7.67E-03

YOL148C	SPT20	0.79857739	8.93340455	2.97E-05	2.45E-03
YLL046C	RNP1	0.80467118	7.90104163	4.67E-05	3.32E-03
YOL147C	PEX11	0.80665244	7.1662519	4.06E-05	3.02E-03
YFL042C	LAM5	0.80738629	9.12416147	2.08E-05	2.00E-03
YNL313C	EMW1	0.80804871	9.39440944	2.15E-05	2.00E-03
YGL241W	KAP114	0.80919847	9.62490835	1.94E-05	2.00E-03
YPL262W	FUM1	0.8141403	8.59910189	2.02E-05	2.00E-03
YAL053W	FLC2	0.8183398	9.50862782	1.53E-05	2.00E-03
YIL157C	COA1	0.81860003	7.64837392	4.05E-05	3.02E-03
YDL227C	HO	0.8220826	9.1359544	1.87E-05	2.00E-03
YHL029C	OCA5	0.82554052	9.26239763	1.46E-05	2.00E-03
YEL056W	HAT2	0.82597891	8.79378705	1.53E-05	2.00E-03
YLL045C	RPL8B	0.82750012	7.43089854	1.99E-05	2.00E-03
YCL044C	MGR1	0.82800375	8.84002577	1.40E-05	2.00E-03
YBL091C	MAP2	0.82832852	8.52566491	1.44E-05	2.00E-03
YKL208W	CBT1	0.82945289	8.28348178	1.57E-05	2.00E-03
YOL111C	MDY2	0.83484137	5.58018707	5.80E-05	3.75E-03
YCL005W	LDB16	0.84733698	6.36279392	2.82E-05	2.42E-03
YBL092W	RPL32	0.84826842	6.40109556	1.17E-05	2.00E-03
YOL077C	BRX1	0.85921944	3.67543432	0.00117967	3.48E-02

Supplementary table 3. Effect of simultaneous αSyn-GFP overexpression and genipin treatment on yeast transcriptome. 437 genes differentially expressed (FDR<0.05) in cells overexpressing αSyn-GFP incubated with 10 µM of genipin comparing with cells carrying the empty vector. Common genes also found differentially expressed in cells overexpressing αSyn-GFP comparing with cells carrying the empty vector are identified by grey shadowing.

ORF	ID	logFC	logCPM	PValue	FDR
YBL014C	RRN6	-7.8007269	1.56230126	3.20E-09	1.33E-07
YFR023W	PES4	-7.8007269	1.56230126	3.20E-09	1.33E-07
YPL183C	RTT10	-7.8007269	1.56230126	3.20E-09	1.33E-07
YCR039C	MATALPHA2	-7.6944813	1.45366362	1.08E-08	3.77E-07
YGL161C	YIP5	-7.6944813	1.45366362	1.08E-08	3.77E-07
YIL087C	AIM19	-7.6944813	1.45366362	1.08E-08	3.77E-07
YLR028C	ADE16	-7.6944813	1.45366362	1.08E-08	3.77E-07
YOL069W	NUF2	-7.6944813	1.45366362	1.08E-08	3.77E-07
YHR062C	RPP1	-6.7121903	13.300162	8.38E-18	9.89E-16
YNL225C	CNM67	-6.6417552	13.3017394	6.49E-19	8.69E-17
YDL133W	SRF1	-6.636548	13.3014541	6.55E-19	8.69E-17
YJL106W	IME2	-6.5602552	13.3032876	1.35E-22	2.39E-20
YCR057C	PWP2	-6.4506474	13.3054	4.25E-25	9.03E-23
YGL150C	INO80	-6.1480073	13.3109891	2.73E-28	7.24E-26
YBL005W-B	YBL005W-B	-6.0583545	13.3124275	4.37E-30	1.33E-27
YNL222W	SSU72	-5.9599268	0.14262222	0.00013144	0.00119397
YCL058W-A	ADF1	-5.8980187	6.46388847	6.06E-47	3.22E-44
YER022W	SRB4	-5.5463169	1.56359541	1.78E-09	8.40E-08
YDL130W-A	STF1	-5.316648	-0.2842315	0.00305255	0.01628281

YJL170C	ASG7	-4.8994657	7.82791022	1.92E-54	1.36E-51
YGL226W	MTC3	-4.5047436	7.70865718	8.62E-96	1.83E-92
YCL058C	FYV5	-4.0226055	6.55312856	6.12E-33	2.60E-30
YPL252C	YAH1	-3.8230933	7.26179163	1.48E-75	1.57E-72
YHR047C	AAP1	-3.3988556	1.90221063	2.12E-07	5.85E-06
YFL065C	YFL065C	-2.6779762	0.766189	0.00098981	0.00636776
YBL108C-A	PAU9	-2.4802212	1.87988346	7.59E-07	1.81E-05
YNL284C-A	YNL284C-A	-2.3820132	8.10130355	1.32E-20	2.16E-18
YFL067W	YFL067W	-2.3415628	1.65940434	1.89E-06	3.94E-05
YNL240C	NAR1	-2.3311961	2.19090964	1.71E-05	0.00023922
YKL217W	JEN1	-2.2218308	7.04436761	4.09E-20	6.21E-18
YOL157C	IMA2	-2.0138326	7.04665186	1.56E-16	1.75E-14
YIL131C	FKH1	-1.7946241	8.23234566	1.97E-13	1.82E-11
YLL021W	SPA2	-1.7713695	8.36757183	1.04E-13	1.05E-11
YEL017C-A	PMP2	-1.6805759	3.58327907	6.61E-06	0.00010799
YGL259W	YPS5	-1.6692256	4.37495575	0.00137899	0.0083407
YPL282C	PAU22	-1.661296	3.9282983	0.00072559	0.00490579
YCL073C	GEX1	-1.6208447	5.79720365	2.00E-05	0.0002647
YHR007C-A	YHR007C-A	-1.5712386	4.01771251	2.62E-09	1.18E-07
YHL048W	COS8	-1.5685005	4.85130421	1.50E-05	0.00021336
YFL062W	COS4	-1.5684521	4.88204913	1.95E-05	0.00026332
YPL274W	SAM3	-1.5544931	7.15373718	7.97E-12	5.98E-10
YHL003C	LAG1	-1.547919	8.2881577	1.50E-10	9.38E-09
YAL067C	SEO1	-1.5373728	6.19606938	0.0002347	0.00189322
YML132W	COS3	-1.5241988	5.46818802	0.00014535	0.00130016
YPL237W	SUI3	-1.4750747	8.29798281	1.32E-09	6.50E-08
YKL183C-A	YKL183C-A	-1.4254412	2.4547566	5.05E-05	0.00056394
YOL164W	BDS1	-1.4204628	5.77295794	5.53E-05	0.00060573
YAL063C-A	YAL063C-A	-1.4029881	3.08537243	4.92E-05	0.00055521
YBL021C	HAP3	-1.4016134	2.4371139	6.06E-05	0.00064351
YGL261C	PAU11	-1.3654694	2.31782473	0.0011346	0.00708457
YNL336W	COS1	-1.3480547	5.21575336	0.0017627	0.01019676
YEL073C	YEL073C	-1.3280246	3.95208115	0.00146038	0.00875815
YHL048C-A	YHL048C-A	-1.2998758	1.32164472	0.00882685	0.03703437
YJL146W	IDS2	-1.2692776	8.05310133	1.15E-13	1.11E-11
YCL074W	YCL074W	-1.2602379	2.34489504	0.00285284	0.01529438
YBL107C	MIX23	-1.2512424	2.21385395	0.0096795	0.04005194
YLL058W	YLL058W	-1.1925655	7.303668	2.71E-07	7.28E-06
YLL066W-B	YLL066W-B	-1.190689	1.7334511	0.0075079	0.03272952
YHL038C	CBP2	-1.181061	7.32686299	3.48E-07	8.75E-06
YDL247W	MPH2	-1.1752029	5.63347211	6.20E-05	0.0006534
YGL263W	COS12	-1.1608812	2.6757599	0.00184646	0.01062343

YML131W	YML131W	-1.1586006	2.68109195	0.00943665	0.03928239
YKL221W	MCH2	-1.1430875	5.17981029	0.0001547	0.00137051
YPL169C	MEX67	-1.1164987	2.24954636	0.00519592	0.02445884
YER038W-A	FMP49	-1.0948932	3.40478129	0.00047006	0.00341759
YFL038C	YPT1	-1.0891513	3.81837215	1.45E-05	0.00021077
YEL076C	YEL076C	-1.0846956	2.1704396	0.01230505	0.047758
YCR063W	BUD31	-1.0715187	3.2752848	0.00064318	0.00441898
YPL170W	DAP1	-1.0715187	3.2752848	0.00064318	0.00441898
YGL235W	YGL235W	-1.0580344	3.55293215	8.70E-05	0.00085886
YFL053W	DAK2	-1.0559641	7.38394641	1.50E-06	3.33E-05
YHL006C	SHU1	-1.0534391	3.67248831	0.00016866	0.00146147
YCL055W	KAR4	-1.0354446	4.09720581	5.94E-05	0.00063732
YEL034W	HYP2	-1.03036	4.13822656	0.00033559	0.00253546
YGL207W	SPT16	-1.0215597	8.62485834	4.85E-06	8.51E-05
YIL134C-A	YIL134C-A	-1.0085505	3.60580783	0.00012327	0.00113294
YCL069W	VBA3	-0.9922784	3.59333382	0.00258387	0.01404897
YIL169C	CSS1	-0.9911277	7.53786241	3.21E-06	6.31E-05
YPL179W	PPQ1	-0.9875555	3.76669747	0.00024652	0.00196019
YDL240W	LRG1	-0.9798301	7.57796661	3.70E-06	7.07E-05
YCL028W	RNQ1	-0.9791539	5.66131879	1.78E-05	0.0002469
YIL098C	FMC1	-0.9591803	3.46795905	0.00221921	0.01233344
YFR028C	CDC14	-0.9583762	3.81912477	0.00026296	0.00206766
YLL023C	POM33	-0.9565328	4.66664471	0.0001155	0.00107572
YHL047C	ARN2	-0.9514667	5.731186	0.00466737	0.02278847
YCR045C	RRT12	-0.9418748	3.6454452	0.00062439	0.00434615
YHR063C	PAN5	-0.9341073	3.16592755	0.00061208	0.00431145
YCR006C	YCR006C	-0.9303643	5.47611046	1.56E-06	3.36E-05
YNL284C-B	YNL284C-B	-0.9261842	8.91608417	5.54E-06	9.49E-05
YGL254W	FZF1	-0.9089848	4.40130999	0.00015669	0.00138031
YJL158C	CIS3	-0.9028415	5.98770201	1.27E-06	2.91E-05
YGL157W	ARI1	-0.8464787	3.14773059	0.00476891	0.02298863
YDL246C	SOR2	-0.8393341	5.15857333	0.0114095	0.04502301
YLR033W	RSC58	-0.8358683	3.51030818	0.00198968	0.01132462
YBL038W	MRPL16	-0.8339512	4.67498304	0.00020767	0.00172058
YCL059C	KRR1	-0.8269073	4.463151	0.00087478	0.00567936
YOL133W	HRT1	-0.8127061	3.91184837	0.01041048	0.04230028
YKL125W	RRN3	-0.8073259	4.03624326	0.00201394	0.01137124
YEL033W	MTC7	-0.8001159	3.90638479	0.00121423	0.00751551
YER027C	GAL83	-0.7980774	3.33476253	0.00521223	0.02448136
YJL113W	YJL113W	-0.7869257	3.41093725	0.00563926	0.0261614
YKL142W	MRP8	-0.7721591	4.09226787	0.00615902	0.02799401
YGL189C	RPS26A	-0.7697884	4.07145694	0.00201182	0.01137124

YLL053C	YLL053C	-0.7648657	8.32507884	9.91E-06	0.00015033
YGL200C	EMP24	-0.7567387	6.03391033	3.68E-05	0.00043433
YJL144W	ROQ1	-0.7486934	3.46416223	0.00892435	0.03736959
YDL189W	RBS1	-0.7338195	7.26395679	8.79E-06	0.00013618
YLL014W	EMC6	-0.7198877	4.00040237	0.00828748	0.03525917
YCR018C	SRD1	-0.7013371	5.0058663	0.00153951	0.00910414
YLR018C	POM34	-0.6944818	4.55939148	0.00928921	0.03882085
YCL020W	YCL020W	-0.6924734	5.91633689	0.00122927	0.00758643
YHR008C	SOD2	-0.6913619	6.47083137	4.64E-05	0.00053226
YOL054W	PSH1	-0.6877514	4.02795224	0.007555	0.03286733
YEL035C	UTR5	-0.682668	4.81416899	0.00413455	0.02075093
YLL013C	PUF3	-0.6758932	8.17576981	1.90E-05	0.00025896
YOL118C	YOL118C	-0.6709024	4.0872637	0.01002222	0.0412138
YBL082C	ALG3	-0.6591982	6.18599413	0.00112547	0.00704827
YEL017W	GTT3	-0.6589618	6.73863356	8.07E-05	0.00080814
YBL051C	PIN4	-0.656551	7.85117826	3.95E-05	0.00046359
YGL208W	SIP2	-0.656359	5.64063019	0.00077578	0.00516296
YHL033C	RPL8A	-0.6453521	8.97332327	0.00023543	0.00189322
YLL028W	TPO1	-0.6438463	6.41734352	0.00205687	0.01155222
YGL248W	PDE1	-0.6427933	9.15878518	0.00019052	0.00161149
YOL124C	TRM11	-0.6414239	5.68920896	0.00257066	0.0140296
YEL069C	HXT13	-0.6376184	4.99566398	0.00566151	0.02618605
YGL191W	COX13	-0.6356762	4.91457852	0.00421671	0.02106371
YNL300W	TOS6	-0.6292579	6.63794611	0.00706716	0.03095538
YGL225W	VRG4	-0.6288409	7.32143099	0.01003652	0.0412138
YLL052C	AQY2	-0.6250749	8.36790074	0.00056353	0.00400126
YDL173W	PAR32	-0.6242446	5.50742531	0.00180966	0.01043996
YNL274C	GOR1	-0.6212238	6.00247369	0.00174332	0.01011223
YHL025W	SNF6	-0.6193526	4.78873236	0.00942143	0.03928239
YIL134W	FLX1	-0.6171082	5.26234319	0.00501653	0.02387914
YJL161W	FMP33	-0.6146726	4.5980355	0.00564006	0.0261614
YER039C	HVG1	-0.6124634	4.41704025	0.01065171	0.04291001
YEL006W	YEA6	-0.6101679	6.36196244	0.00112319	0.00704827
YDL234C	GYP7	-0.6096381	10.0835254	0.00225272	0.01244785
YKL212W	SAC1	-0.6092767	9.95761689	0.00155307	0.0091334
YJL203W	PRP21	-0.6089625	4.19235121	0.00823614	0.03518173
YJL111W	CCT7	-0.60715	4.10584108	0.00852046	0.03603373
YFL050C	ALR2	-0.6060013	9.68913035	0.00952541	0.03957427
YJL212C	OPT1	-0.6036555	10.0282189	0.00363424	0.01859153
YBL075C	SSA3	-0.599397	6.48582014	0.00348543	0.0181362
YEL062W	NPR2	-0.5946747	9.85555888	0.00250424	0.01373771
YAL060W	BDH1	-0.5928396	9.59671597	0.00140806	0.00848616

YPL240C	HSP82	-0.5872838	6.70627452	0.00132467	0.00808127
YML079W	YML079W	-0.5806879	5.24388502	0.00701116	0.03088111
YGL193C	YGL193C	-0.5795432	8.49091872	0.00027213	0.00212404
YEL064C	AVT2	-0.5772734	5.31915579	0.0111074	0.04420317
YML092C	PRE8	-0.5750998	4.82045453	0.00564386	0.0261614
YNL270C	ALP1	-0.5749989	7.92356862	0.00225738	0.01244785
YFL002W-B	YFL002W-B	-0.5716851	11.030851	0.00700081	0.03088111
YFL056C	AAD6	-0.5714189	4.72965042	0.00804075	0.03455569
YJL128C	PBS2	-0.5700351	5.75126313	0.01030853	0.04208654
YKL163W	PIR3	-0.5664221	6.30095694	0.00235654	0.01296097
YAL017W	PSK1	-0.5656135	8.52352258	0.0004291	0.00317414
YCR005C	CIT2	-0.5646524	6.40015804	0.00346933	0.01809676
YGL209W	MIG2	-0.5630408	6.81373815	0.00079264	0.00520981
YDL175C	AIR2	-0.5607631	6.46709103	0.00117838	0.00733634
YGL198W	YIP4	-0.559726	6.53163589	0.00079617	0.00521686
YML086C	ALO1	-0.5499071	7.17517668	0.00107415	0.00682763
YHR037W	PUT2	-0.5474913	6.90994571	0.00466407	0.02278847
YNL321W	VNX1	-0.5462775	9.97753958	0.00380137	0.01921503
YML117W	NAB6	-0.5423851	10.1571292	0.00618426	0.02799401
YCL051W	LRE1	-0.5365105	8.83685235	0.00171745	0.00998941
YPL268W	PLC1	-0.5341124	9.77382423	0.00358877	0.01849264
YFL021W	GAT1	-0.5284302	6.9917143	0.00224791	0.01244785
YML052W	SUR7	-0.5199712	6.11458494	0.00550932	0.02576276
YNL269W	BSC4	-0.5134098	7.42785235	0.00368299	0.01870572
YML063W	RPS1B	-0.51121	5.10518471	0.01059278	0.04275374
YOL097C	WRS1	-0.5030867	8.11298318	0.00487835	0.02337863
YFL008W	SMC1	-0.4975295	7.82881317	0.00508809	0.02416557
YLL024C	SSA2	-0.4975021	7.51908817	0.00356092	0.01839373
YIL114C	POR2	-0.4829651	5.54550414	0.01140057	0.04502301
YEL002C	WBP1	-0.4812485	6.22778197	0.00665428	0.02972178
YDL203C	ACK1	-0.480514	7.01056552	0.00658734	0.02950404
YIL119C	RPI1	-0.4717208	8.22579842	0.00760897	0.03303446
YLL006W	MMM1	-0.4646635	8.23667262	0.00843379	0.03573841
YLL001W	DNM1	-0.4645982	7.09123479	0.00513312	0.02427085
YNL286W	CUS2	-0.4628773	6.56816851	0.00608142	0.02780867
YAL026C	DRS2	-0.4575289	7.96916494	0.00707177	0.03095538
YOL103W-B	YOL103W-B	-0.4506722	8.11840769	0.00644143	0.02897278
YPL214C	THI6	-0.4395302	6.68899183	0.0102534	0.04194214
YDL160C	DHH1	-0.4371668	6.71046367	0.01044058	0.04230028
YHL007C	STE20	-0.4276654	7.90559445	0.00969698	0.04005194
YJL141C	YAK1	-0.4063578	7.36138676	0.01162261	0.04569408
YOL138C	RTC1	0.40609674	9.2064191	0.01032893	0.04208889

YAL039C	CYC3	0.41354104	8.83598099	0.01245339	0.04815764
YML104C	MDM1	0.42825284	8.77916471	0.00481556	0.02312995
YKL171W	NNK1	0.42933008	8.8128299	0.00518701	0.02445884
YML045W-A	YML045W-A	0.43635716	6.87257429	0.00684182	0.0302608
YIL152W	YIL152W	0.43643397	6.07161922	0.01183072	0.04608555
YBL019W	APN2	0.43700515	7.18640312	0.00658044	0.02950404
YPL247C	YPL247C	0.43862012	7.09655452	0.01043641	0.04230028
YJL176C	SWI3	0.44254915	7.36882082	0.00674249	0.03000901
YML123C	PHO84	0.44895754	6.24431261	0.01279566	0.04930161
YAL055W	PEX22	0.44965671	6.41126172	0.01071697	0.04300969
YFL037W	TUB2	0.45550728	7.15529382	0.00490164	0.02343736
YFL004W	VTC2	0.4557994	8.41739981	0.0047222	0.02294102
YIL123W	SIM1	0.45674242	8.15904531	0.00258745	0.01404897
YBL084C	CDC27	0.46076671	10.2214491	0.01295918	0.04975107
YDL149W	ATG9	0.46706966	7.05497123	0.00346606	0.01809676
YFL036W	RPO41	0.46755661	9.28317519	0.00361798	0.01855307
YER016W	BIM1	0.47761397	6.14526085	0.00681025	0.03024719
YNL307C	MCK1	0.47832429	6.65293659	0.00451805	0.02230655
YHL032C	GUT1	0.47846509	6.55533598	0.01243019	0.04815563
YHL023C	NPR3	0.48666341	9.0333552	0.00193348	0.01109398
YGL228W	SHE10	0.4871154	9.38449168	0.00471912	0.02294102
YGL219C	MDM34	0.48756356	6.9361616	0.00458063	0.02251083
YBL017C	PEP1	0.48829841	8.16736358	0.0012472	0.00767483
YML106W	URA5	0.48991147	6.27475524	0.00476877	0.02298863
YEL061C	CIN8	0.49064027	7.29358091	0.00617776	0.02799401
YNL287W	SEC21	0.49376196	7.50794382	0.00203596	0.0114651
YDL179W	PCL9	0.49647836	6.16202817	0.00343666	0.01801487
YHR026W	VMA16	0.49818185	9.05928235	0.01148656	0.04524297
YIL162W	SUC2	0.49867337	6.11083372	0.01070364	0.04300969
YAL058W	CNE1	0.49966137	6.05731889	0.01113105	0.04420317
YDL161W	ENT1	0.50221407	6.72082187	0.00799051	0.03440942
YEL022W	GEA2	0.50342607	8.39623571	0.00261846	0.01418112
YLL008W	DRS1	0.50389804	8.66212752	0.00255658	0.01398871
YLR024C	UBR2	0.50434988	8.43059585	0.00078146	0.00516379
YFL011W	HXT10	0.50603371	7.39541207	0.00284977	0.01529438
YPL242C	IQG1	0.51022022	8.16224958	0.00160831	0.00943214
YDL165W	CDC36	0.51076226	9.71697991	0.00666395	0.02972178
YJL134W	LCB3	0.51275258	6.41203409	0.00220755	0.01230085
YAL042W	ERV46	0.51302548	8.43659868	0.0027809	0.01498442
YJL130C	URA2	0.51650303	10.1933694	0.00340686	0.01790287
YIL150C	MCM10	0.51678194	8.7052935	0.00339618	0.01789106
YAL046C	BOL3	0.52447294	5.75080387	0.00633921	0.02857355

YNL267W	PIK1	0.52663568	13.2576619	0.00864615	0.0364926
YJL198W	PHO90	0.52885464	8.90483909	0.00126247	0.00774631
YOL096C	COQ3	0.55145834	7.46431054	0.00063029	0.00437292
YFR019W	FAB1	0.55640007	8.64756451	0.00020764	0.00172058
YCL048W	SPS22	0.55663602	8.06242464	0.0036647	0.01870229
YDL132W	CDC53	0.55684336	5.02140237	0.0049136	0.02344173
YFL014W	HSP12	0.55751347	6.06818997	0.00141103	0.00848616
YGL167C	PMR1	0.5589598	7.78170893	0.0002217	0.00181728
YML114C	TAF8	0.56013154	8.09250722	0.0035109	0.01822406
YGL233W	SEC15	0.56186277	8.16160941	0.00021089	0.00173538
YBL086C	YBL086C	0.56352064	8.61298186	0.00153787	0.00910414
YFL015C	YFL015C	0.56429789	7.2194768	0.00196462	0.01124229
YGL253W	HXK2	0.57278648	6.10885952	0.00151533	0.00903666
YJL117W	PHO86	0.57399393	4.8961694	0.00586491	0.02695064
YHR018C	ARG4	0.57648437	13.7220527	0.01112931	0.04420317
YNL282W	POP3	0.58250397	6.61478567	0.00074993	0.00503831
YBL055C	YBL055C	0.58431463	5.92181439	0.01049556	0.04244204
YBL069W	AST1	0.58754337	6.14854292	0.01285595	0.04944419
YKL138C	MRPL31	0.58936652	4.5762154	0.00512018	0.02426372
YEL036C	ANP1	0.59164532	6.85566268	0.00025272	0.00200193
YOL112W	MSB4	0.59238973	8.26170366	0.00063862	0.00441623
YJL165C	HAL5	0.59461512	8.5994986	0.00080593	0.0052646
YHR014W	SPO13	0.59748797	6.11632573	0.00048392	0.00349502
YCR008W	SAT4	0.59793309	8.27494658	0.00017543	0.00150179
YBL057C	PTH2	0.59992733	6.35677439	0.00296863	0.01587505
YGL203C	KEX1	0.60017337	7.7322168	0.00023413	0.00189322
YJL126W	NIT2	0.60971144	6.37456461	0.00066815	0.00454644
YLR007W	NSE1	0.61241019	10.7725524	0.00682931	0.0302608
YGL206C	CHC1	0.61413865	8.87731484	0.00055666	0.00396574
YJL166W	QCR8	0.61485631	6.67938149	0.00069911	0.00474189
YJL131C	AIM23	0.61645191	10.8027523	0.00582722	0.02683556
YOL055C	THI20	0.61689831	4.32373311	0.00775673	0.03353876
YER037W	PHM8	0.63212705	4.14841853	0.00360501	0.01853134
YML076C	WAR1	0.63293438	7.87452625	0.00010499	0.00099506
YML066C	SMA2	0.63305907	13.2992487	0.0045746	0.02251083
YNL243W	SLA2	0.63657197	7.7240801	2.44E-05	0.00030608
YKL168C	KKQ8	0.63703981	9.61658201	0.00031164	0.00237117
YGL194C	HOS2	0.63791028	13.3717034	0.00437964	0.02172423
YCL033C	MXR2	0.63955317	4.98998118	0.00338719	0.01788806
YLL010C	PSR1	0.63999279	7.74884981	0.00018121	0.00154497
YGL148W	ARO2	0.6405609	3.24010228	0.01178713	0.04608555
YEL031W	SPF1	0.64536972	7.84016841	0.00018783	0.00159507

YDL148C	NOP14	0.64773468	7.60276867	2.10E-05	0.00027382
YJL155C	FBP26	0.65207664	7.81607458	0.00012231	0.00113294
YEL024W	RIP1	0.65700015	7.13434753	8.85E-05	0.00086964
YEL046C	GLY1	0.65794696	8.20454104	0.00053657	0.00384845
YIL142W	CCT2	0.65890818	6.55246411	7.25E-05	0.00073699
YHL030W	ECM29	0.66051546	9.89144037	0.00041577	0.00308628
YDL230W	PTP1	0.66087687	7.61869001	0.00310625	0.01652772
YIL128W	MET18	0.6649263	7.44534427	0.00046467	0.00340639
YKL178C	STE3	0.6663361	7.10868585	7.05E-05	0.00072691
YNL271C	BNI1	0.66677008	10.4783718	0.00019676	0.00164461
YHR001W	OSH7	0.66767322	8.54253104	0.00026637	0.00208674
YAL044W-A	BOL1	0.67034872	6.24707408	4.45E-05	0.00051677
YAL013W	DEP1	0.67048752	7.71324253	7.29E-05	0.00073699
YBL061C	SKT5	0.67125122	8.60620008	0.00019563	0.00164162
YNL254C	RTC4	0.67284049	6.41580755	0.00031273	0.00237117
YHR064C	SSZ1	0.67735617	4.57549917	0.00075734	0.0050561
YPL232W	SSO1	0.68016893	7.45543607	4.87E-05	0.00055258
YCL001W-A	YCL001W-A	0.68028479	5.62792022	0.00082825	0.00539379
YJL154C	VPS35	0.68103829	9.85694917	0.00010239	0.00098358
YEL013W	VAC8	0.68234801	8.04022845	2.73E-05	0.00033655
YAR071W	PHO11	0.68367452	4.62474767	0.0007288	0.00491185
YER015W	FAA2	0.68567647	6.77296854	5.52E-05	0.00060573
YAL059W	ECM1	0.68815011	5.36612103	0.00352922	0.01827445
YOL136C	PFK27	0.68821939	8.47644453	0.00014575	0.00130016
YDL197C	ASF2	0.68961159	7.16494509	0.0001316	0.00119397
YLR023C	IZH3	0.68983684	5.09914838	0.00219571	0.01226708
YML081W	TDA9	0.69136607	9.24251851	7.60E-05	0.00076454
YFL046W	FMP32	0.69212155	7.11368916	0.00166771	0.00972678
YHR005C	GPA1	0.6952547	6.925134	6.77E-05	0.00070405
YLL019C	KNS1	0.69674336	8.95517075	7.28E-05	0.00073699
YCR061W	YCR061W	0.69914617	4.53148391	0.00056731	0.00401468
YPL202C	AFT2	0.69969324	6.4462313	9.49E-05	0.00092405
YOL139C	CDC33	0.70005726	5.75614391	8.96E-05	0.00087614
YNL278W	CAF120	0.70254951	7.78883119	0.00011477	0.00107572
YIL137C	TMA108	0.70301973	7.50857668	0.00030057	0.00230363
YPL230W	USV1	0.70428079	6.06539856	0.0070406	0.03094656
YLL016W	SDC25	0.70465784	8.04732608	4.57E-05	0.00052712
YDL206W	YDL206W	0.70608603	7.30440825	0.00065537	0.00447377
YLL004W	ORC3	0.70721231	12.6628647	0.00029772	0.00229004
YJL125C	GCD14	0.70962366	4.7039603	0.00631522	0.028526
YIL117C	PRM5	0.71577041	13.0277457	0.00108221	0.00685828
YBL047C	EDE1	0.71584148	10.5560523	5.04E-05	0.00056394

YLL046C	RNP1	0.71595888	7.87401068	6.00E-06	0.00010176
YAR008W	SEN34	0.7173776	5.57294122	8.34E-05	0.00083169
YNL283C	WSC2	0.71801411	7.81106773	6.04E-05	0.00064351
YOL075C	YOL075C	0.72024989	7.76944399	4.27E-06	7.61E-05
YKL208W	CBT1	0.72045368	8.24531271	6.69E-06	0.00010836
YAL023C	PMT2	0.72773969	9.10694913	3.62E-05	0.00042927
YML058W	SML1	0.72888947	4.26419017	0.00150031	0.00897228
YNL224C	SQS1	0.72979393	4.9998569	0.00016164	0.00141223
YFL023W	BUD27	0.73739539	7.26188455	2.27E-05	0.00028877
YFL005W	SEC4	0.74222654	7.49850264	2.23E-05	0.00028561
YJL162C	JJJ2	0.74303901	6.71949438	0.00012326	0.00113294
YIL107C	PFK26	0.74722296	12.1832722	0.00112432	0.00704827
YCL008C	STP22	0.74812645	8.03573317	3.53E-05	0.00042126
YHR005C-A	TIM10	0.74926929	4.15244813	0.00880302	0.03700753
YJL206C	YJL206C	0.75145034	8.49520026	1.60E-06	3.40E-05
YBL056W	PTC3	0.75285836	6.5434168	6.22E-05	0.0006534
YKL134C	Oct-01	0.75425297	7.10686634	3.91E-06	7.22E-05
YPL233W	NSL1	0.75543208	7.96297073	3.01E-05	0.00036264
YKL113C	RAD27	0.75608622	3.78686381	0.00430111	0.0213847
YPL222W	FMP40	0.75720008	10.1830037	2.19E-05	0.00028202
YGL196W	DSD1	0.75754488	8.65231168	7.44E-06	0.00011869
YFL010C	WWM1	0.76110534	5.12142856	0.00338068	0.01788806
YGL169W	SUA5	0.76465395	4.20738824	0.00061658	0.00431352
YOL100W	PKH2	0.76497088	10.4070185	1.96E-05	0.00026332
YKL181W	PRS1	0.76701628	7.81710305	3.83E-06	7.13E-05
YAL032C	PRP45	0.7689794	6.30280389	5.02E-06	8.73E-05
YIL157C	COA1	0.77001966	7.64258111	2.11E-06	4.30E-05
YAL020C	ATS1	0.77642482	6.6250985	6.14E-06	0.00010176
YGL212W	VAM7	0.78336275	5.97072215	0.00106357	0.0068011
RUF20	RUF20	0.78495861	8.30199923	3.83E-06	7.13E-05
YPL189W	GUP2	0.79055005	6.94753285	2.03E-06	4.18E-05
YHR039C	MSC7	0.79375631	6.45643672	6.88E-06	0.00011068
YDL183C	YDL183C	0.79953314	9.54835347	4.17E-06	7.54E-05
YCR001W	YCR001W	0.80052545	4.27391745	0.01021153	0.04185151
YFL010W-A	AUA1	0.80594806	3.33124951	0.00197504	0.01127156
YML111W	BUL2	0.81672311	9.23793305	2.75E-07	7.29E-06
YIL092W	YIL092W	0.81691385	6.99569192	7.78E-07	1.84E-05
YML071C	COG8	0.82047595	9.53300609	2.72E-06	5.46E-05
YLL009C	COX17	0.82545991	6.9784044	3.37E-06	6.50E-05
YCL018W	LEU2	0.82726301	6.28417895	0.00016386	0.00142573
YJL121C	RPE1	0.83195865	4.80467651	0.00024612	0.00196019
YJL171C	TOH1	0.8320192	6.53087079	0.00822537	0.03518173

YAL012W	CYS3	0.83608887	9.85372247	1.33E-06	3.01E-05
YOL077C	BRX1	0.83946068	3.66859496	0.0007832	0.00516379
YLL048C	YBT1	0.83949757	9.86432672	1.44E-05	0.00021077
YNL304W	YPT11	0.84240252	6.77479514	8.73E-08	2.65E-06
YDL193W	NUS1	0.84451008	5.76018489	0.000484	0.00349502
YOL105C	WSC3	0.85001311	6.49858643	1.29E-05	0.00019031
YAR028W	YAR028W	0.85182191	5.85437148	1.57E-06	3.36E-05
YDL222C	FMP45	0.85480861	6.86557662	3.36E-06	6.50E-05
YBL033C	RIB1	0.86196916	5.55755106	2.31E-06	4.67E-05
YCR009C	RVS161	0.87847049	9.17325805	1.17E-07	3.45E-06
YIL122W	POG1	0.87866781	9.41338418	2.30E-07	6.26E-06
YDL227C	HO	0.88215682	9.18959991	3.09E-08	1.01E-06
YCR015C	CTO1	0.88449139	11.6932073	1.57E-05	0.00022218
YDL177C	YDL177C	0.88454811	11.7299347	1.47E-05	0.0002128
YJL207C	LAA1	0.89392475	10.3100981	2.82E-06	5.59E-05
YHR013C	ARD1	0.8953999	8.94727821	4.69E-08	1.46E-06
YOL149W	DCP1	0.89637775	5.10343883	0.0004692	0.00341759
YEL056W	HAT2	0.8977287	8.85360716	8.44E-09	3.32E-07
YBL041W	PRE7	0.90597651	11.5314053	1.29E-05	0.00019031
YPL204W	HRR25	0.90863962	11.7907934	0.00012957	0.00118571
YPL262W	FUM1	0.91092171	8.67255879	4.76E-09	1.94E-07
YJL105W	SET4	0.91235715	4.71204003	1.13E-05	0.00016956
YER017C	AFG3	0.91394138	7.05960063	6.46E-08	1.99E-06
YOL116W	MSN1	0.91638491	6.72457207	0.0042305	0.02108296
YIL159W	BNR1	0.91789127	9.50978096	2.87E-05	0.00034854
YCR026C	NPP1	0.92320265	12.1311355	0.00010424	0.00099504
YNL313C	EMW1	0.9238707	9.4788967	1.42E-08	4.77E-07
YBL091C	MAP2	0.93049128	8.60229475	2.19E-09	1.01E-07
YCL044C	MGR1	0.930782	8.91720762	1.65E-09	7.96E-08
YHL029C	OCA5	0.93125531	9.34151008	8.65E-09	3.34E-07
YOL083W	ATG34	0.93147751	9.50137936	4.01E-06	7.34E-05
YCL005W	LDB16	0.93179387	6.42490651	5.53E-07	1.33E-05
YAR035W	YAT1	0.93966379	7.10709328	1.33E-08	4.56E-07
YFL042C	LAM5	0.94198534	9.21909687	5.23E-09	2.10E-07
YKL170W	MRPL38	0.94382318	7.63726992	8.86E-09	3.36E-07
YNL281W	HCH1	0.9539673	4.80986052	0.00061331	0.00431145
YOL148C	SPT20	0.97032125	9.04908979	1.25E-09	6.33E-08
YGL241W	KAP114	0.97627741	9.73838255	2.77E-09	1.23E-07
YML083C	YML083C	0.99388232	6.96047979	0.00135409	0.00823705
YAL053W	FLC2	1.00082542	9.63111298	8.29E-10	4.54E-08
YGL149W	YGL149W	1.00291697	2.06084361	0.00477531	0.02298863
YNL256W	FOL1	1.00853653	11.8801246	2.86E-05	0.00034854

YCL035C	GRX1	1.01619828	3.90407907	8.16E-06	0.00012739
YER007W	PAC2	1.0218774	11.886708	2.39E-05	0.00030191
YAR010C	YAR010C	1.03284841	11.7910128	2.00E-05	0.0002647
YKL209C	STE6	1.05724473	9.53673227	1.57E-06	3.36E-05
YHR028C	DAP2	1.0583161	11.6216399	8.99E-06	0.00013827
YDL164C	CDC9	1.06926629	11.6041	7.99E-06	0.00012562
YOL111C	MDY2	1.08977046	5.73904488	3.61E-08	1.16E-06
YAL025C	MAK16	1.10983125	6.87164778	0.0002874	0.00222679
YFR034C	PHO4	1.11122571	3.67170982	7.12E-05	0.00072995
YFR010W	UBP6	1.1133622	11.5128188	4.63E-06	8.18E-05
YLL045C	RPL8B	1.11951108	7.61554859	2.42E-12	1.90E-10
YBL092W	RPL32	1.12803834	6.57874596	9.32E-10	4.95E-08
YKL151C	NNR2	1.13099977	11.5694456	4.19E-06	7.54E-05
YOL147C	PEX11	1.15380449	7.38234366	1.65E-12	1.35E-10
YBL064C	PRX1	1.15926717	6.7481292	9.68E-05	0.00093865
YIL158W	AIM20	1.16184822	7.30766625	5.74E-11	3.81E-09
YML043C	RRN11	1.1642101	6.33559174	9.64E-10	4.99E-08
YAR029W	YAR029W	1.17766708	1.59829085	0.00954646	0.03958423
YPL264C	YPL264C	1.18817183	7.51764029	3.24E-05	0.00038804
YCL047C	POF1	1.19380919	7.16346883	0.00019493	0.00164162
YML054C	CYB2	1.19682765	10.8084466	3.50E-07	8.75E-06
YBL095W	MRX3	1.20372676	7.03618748	2.01E-05	0.0002647
YAL054C	ACS1	1.20770268	9.01881864	5.06E-06	8.73E-05
YDL200C	MGT1	1.20954196	6.39291523	5.82E-05	0.00063007
YGL243W	TAD1	1.2133971	7.81837828	4.69E-05	0.00053501
YFL045C	SEC53	1.21695911	7.06142158	0.00010452	0.00099504
YCL045C	EMC1	1.21733555	8.79528306	8.17E-12	5.98E-10
YNL315C	ATP11	1.2275764	7.31065677	2.06E-05	0.00026983
YPL263C	KEL3	1.23769495	8.45163213	7.93E-11	5.10E-09
YML113W	DAT1	1.24762311	7.73490221	1.85E-05	0.00025548
YDL229W	SSB1	1.25206227	8.35268704	1.42E-06	3.18E-05
YOL151W	GRE2	1.2672964	7.99819935	1.89E-05	0.00025851
YEL058W	PCM1	1.27533733	8.63875607	6.06E-06	0.00010176
YBL093C	ROX3	1.28179568	7.76954691	6.58E-06	0.00010799
YLR008C	PAM18	1.32491306	10.5054427	1.28E-07	3.62E-06
YFL044C	OTU1	1.32905184	7.01909383	1.22E-07	3.54E-06
YNL314W	DAL82	1.37659727	7.72533746	3.17E-07	8.22E-06
YGL242C	YGL242C	1.37943032	6.98211686	1.24E-07	3.55E-06
YEL027W	VMA3	1.40161187	6.03957032	9.97E-07	2.30E-05
YML058W-A	HUG1	1.64635145	1.81421755	8.60E-05	0.00085339

Supplementary table 4. Effect of genipin protection in yeast transcriptome. 235 genes were found differentially expressed (FDR<0.05) in cells overexpressing αSyn-GFP incubated with 10 µM of genipin comparing with cells overexpressing αSyn-GFP.

ORF	ID	logFC	logCPM	PValue	FDR
YBL014C	RRN6	-8.892796	2.26672279	4.56E-23	6.91E-21
YFR023W	PES4	-8.892796	2.26672279	4.56E-23	6.91E-21
YPL183C	RTT10	-8.892796	2.26672279	4.56E-23	6.91E-21
YCR039C	MATALPHA2	-8.8388896	2.21645615	9.63E-22	1.08E-19
YGL161C	YIP5	-8.8388896	2.21645615	9.63E-22	1.08E-19
YIL087C	AIM19	-8.8388896	2.21645615	9.63E-22	1.08E-19
YLR028C	ADE16	-8.8388896	2.21645615	9.63E-22	1.08E-19
YOL069W	NUF2	-8.8388896	2.21645615	9.63E-22	1.08E-19
YNL222W	SSU72	-7.2491367	0.81042776	8.64E-10	3.99E-08
YDL130W-A	STF1	-6.8136361	0.45624609	1.16E-07	4.34E-06
YCL058W-A	ADF1	-6.739753	7.01787076	1.18E-54	8.37E-52
YHR062C	RPP1	-6.5449997	12.8223687	7.76E-14	4.71E-12
YNL225C	CNM67	-6.47409	12.823813	8.16E-15	5.26E-13
YDL133W	SRF1	-6.4691763	12.8238429	8.15E-15	5.26E-13
YJL106W	IME2	-6.3921126	12.8253275	3.50E-18	2.98E-16
YER022W	SRB4	-6.3850078	2.23669602	6.33E-21	6.72E-19
YCR057C	PWP2	-6.2819496	12.8274903	1.51E-20	1.53E-18
YGL150C	INO80	-5.9795898	12.8351881	7.74E-24	1.64E-21
YBL005W-B	YBL005W-B	-5.8908592	12.8381652	6.95E-26	1.64E-23
YHR050W-A	YHR050W-A	-5.5723591	-0.4304251	0.00078361	0.00931632
YJL170C	ASG7	-5.3568841	7.99362672	7.88E-78	1.67E-74
YCL058C	FYV5	-4.838599	7.07319289	1.11E-39	4.72E-37
YGL226W	MTC3	-4.5421655	7.4652347	1.13E-65	1.20E-62
YHR047C	AAP1	-3.9920022	2.42695381	6.56E-18	5.36E-16
YPL252C	YAH1	-3.8436593	7.01795875	1.65E-49	8.78E-47
YOL164W-A	YOL164W-A	-3.4668585	-0.1239227	0.0042807	0.03264044
YKL217W	JEN1	-2.8693191	7.3840816	4.26E-23	6.91E-21
YNL284C-A	YNL284C-A	-2.864488	8.29183776	2.19E-30	7.77E-28
YNL240C	NAR1	-2.8113168	2.57885381	1.29E-12	6.71E-11
YOL157C	IMA2	-2.6830856	7.40288104	2.44E-20	2.35E-18
YIL131C	FKH1	-2.300885	8.44490667	5.81E-20	5.14E-18
YLL021W	SPA2	-2.2362733	8.54493325	5.04E-20	4.66E-18
YPL274W	SAM3	-2.2206252	7.50100383	1.92E-15	1.36E-13
YHL003C	LAG1	-2.0890423	8.52964804	3.89E-16	2.85E-14
YPL237W	SUI3	-2.0381474	8.55727839	2.00E-15	1.37E-13
YLL058W	YLL058W	-1.8323715	7.62476985	1.09E-10	5.28E-09
YHL038C	CBP2	-1.8084721	7.63816075	2.81E-10	1.32E-08
YKL183C-A	YKL183C-A	-1.7897304	2.61020028	3.91E-07	1.34E-05

YFL053W	DAK2	-1.6715928	7.68430928	1.19E-09	5.36E-08
YHL048C-A	YHL048C-A	-1.5875777	1.41429469	0.00078476	0.00931632
YGL207W	SPT16	-1.5869964	8.88359755	1.94E-11	9.81E-10
YJL146W	IDS2	-1.5812738	8.11839541	3.38E-17	2.57E-15
YER038W-A	FMP49	-1.5634997	3.61631217	1.59E-05	0.00034841
YIL169C	CSS1	-1.5508951	7.79495446	7.71E-09	3.21E-07
YDL240W	LRG1	-1.5242787	7.82337973	7.57E-09	3.21E-07
YPL169C	MEX67	-1.5191562	2.44164332	0.00121925	0.01257722
YPL170W	DAP1	-1.5122255	3.46264134	3.08E-05	0.00062657
YCR063W	BUD31	-1.4938267	3.44943882	4.26E-05	0.00081637
YNL284C-B	YNL284C-B	-1.4230727	9.12323293	1.30E-09	5.77E-08
YLL066W-B	YLL066W-B	-1.325336	1.75051665	0.00131837	0.01315278
YFL038C	YPT1	-1.3179478	3.87288295	7.88E-06	0.00019946
YHL006C	SHU1	-1.2631136	3.71558029	4.25E-05	0.00081637
YJL113W	YJL113W	-1.2445875	3.62804687	5.34E-07	1.72E-05
YOL054W	PSH1	-1.2394169	4.29985769	9.58E-06	0.00023134
YHR007C-A	YHR007C-A	-1.2085983	3.62752805	7.05E-06	0.00018737
YBL039W-B	MIN6	-1.1884219	3.9214898	5.25E-07	1.71E-05
YCL005W-A	VMA9	-1.18473	2.97289056	0.0019535	0.01804864
YLL023C	POM33	-1.1599077	4.69201063	3.96E-06	0.00011233
YGL235W	YGL235W	-1.1100457	3.4791607	0.0008915	0.01002352
YEL076C	YEL076C	-1.1046988	2.08645453	0.00492197	0.03613671
YER039C	HVG1	-1.0980124	4.6382498	2.39E-05	0.00049919
YPL179W	PPQ1	-1.0917728	3.73463819	3.96E-05	0.00078581
YCR045C	RRT12	-1.0885345	3.64211043	0.00010066	0.00176783
YAL063C-A	YAL063C-A	-1.0879044	2.75411826	0.00281701	0.02384916
YIL071C	PCI8	-1.0746677	4.15226812	0.00011683	0.00200208
YGL146C	RRT6	-1.0595794	4.04327631	0.00011806	0.00200534
YJL103C	GSM1	-1.0563413	2.8437638	0.00554277	0.0386374
YBL021C	HAP3	-1.0304082	2.07727142	0.00646287	0.04351585
YEL020W-A	TIM9	-1.0260412	3.57198403	0.00010048	0.00176783
YML129C	COX14	-0.965136	2.52166089	0.00445929	0.03384285
YFR028C	CDC14	-0.9570029	3.71750677	0.00021633	0.00343059
YPL229W	YPL229W	-0.9469107	4.87895257	6.80E-06	0.00018288
YJL161W	FMP33	-0.9445684	4.71492833	2.99E-05	0.00061617
YIL127C	RRT14	-0.9352162	4.85566161	8.53E-06	0.0002107
YLR040C	AFB1	-0.9276222	3.37292779	0.00211733	0.0193104
YHL048W	COS8	-0.8905543	4.23063324	0.00085625	0.00983534
YFL062W	COS4	-0.8799681	4.25507693	0.00140979	0.01393401
YGL193C	YGL193C	-0.8787838	8.569843	2.06E-05	0.00044147
YCL026C-B	HBN1	-0.8718383	4.64203197	1.28E-05	0.00029476
YKL125W	RRN3	-0.8496941	3.96601145	0.00124714	0.01273553

YBL071W-A	KTI11	-0.847705	3.12848377	0.00466982	0.03471052
YHR065C	RRP3	-0.8445982	5.0033953	0.00098641	0.01069448
YBL004W	UTP20	-0.8240721	5.32212483	0.0010101	0.01084073
YML039W	YML039W	-0.8168801	4.78617846	4.15E-05	0.00081637
RPR1	RPR1	-0.8139803	4.5682578	0.00155724	0.01517948
YCL028W	RNQ1	-0.8085764	5.42607014	0.00017679	0.0028898
YFL016C	MDJ1	-0.8075566	5.07197543	0.00023416	0.00365876
YIL134C-A	YIL134C-A	-0.8050263	3.37158381	0.00254242	0.02210198
YFR037C	RSC8	-0.7757189	5.29676277	0.00189638	0.01759744
YEL034W	HYP2	-0.7722921	3.85422997	0.00232544	0.02076286
YKL182W	FAS1	-0.771222	9.51340751	0.00045272	0.00632915
YBL038W	MRPL16	-0.7682211	4.52867662	0.00092114	0.01030219
YJL111W	CCT7	-0.7659254	4.1164463	0.00089007	0.01002352
YLL003W	SFI1	-0.7599094	9.37724916	0.00055557	0.00742503
YNL223W	ATG4	-0.7499974	4.78217443	0.0051098	0.03687391
YDL202W	MRPL11	-0.74015	4.4877736	0.00709722	0.04698313
YGL208W	SIP2	-0.7258128	5.58117871	0.00012463	0.00208532
YJL172W	CPS1	-0.7210683	5.79531187	0.00155584	0.01517948
YFL002W-B	YFL002W-B	-0.7084555	11.0058015	0.0018261	0.01724648
YCL073C	GEX1	-0.7077043	4.99183413	0.00074459	0.00909337
YOL104C	NDJ1	-0.6976874	5.46583196	0.00043527	0.00616639
YIL134W	FLX1	-0.6887222	5.20876177	0.00157209	0.01525428
YJL179W	PFD1	-0.6835322	4.49692193	0.00125258	0.01273553
YOL117W	RRI2	-0.6762273	5.99385488	0.00274318	0.02341068
YNL269W	BSC4	-0.6712011	7.42473332	0.00119199	0.01235604
YCR002C	CDC10	-0.6702843	5.98422906	0.00234769	0.0207868
YGL191W	COX13	-0.6651655	4.83680822	0.00056788	0.00754213
YOL164W	BDS1	-0.6536571	5.09627747	0.00388547	0.03046723
YKL129C	MYO3	-0.6535652	5.10428149	0.00085497	0.00983534
YOL113W	SKM1	-0.6464082	5.76051891	0.00550945	0.0386374
YCL032W	STE50	-0.636127	6.25456404	0.00481111	0.03562234
YNL277W	MET2	-0.6313979	6.14353046	0.00169801	0.01625345
YNL300W	TOS6	-0.6302286	6.53793798	0.0026963	0.02310335
YFL009W	CDC4	-0.6171454	6.83445889	0.00366889	0.02919993
YPL240C	HSP82	-0.6073055	6.61551443	0.00110596	0.01163444
YEL006W	YEA6	-0.5954138	6.25021397	0.00060089	0.00773872
YIL161W	YIL161W	-0.5921046	5.81810061	0.00096952	0.01061969
YDL175C	AIR2	-0.5915185	6.38607028	0.00061325	0.00780328
YCR022C	YCR022C	-0.5906162	5.27234219	0.00224595	0.02013774
YML063W	RPS1B	-0.5793682	5.05476546	0.00520779	0.03726113
YKL163W	PIR3	-0.5782992	6.2095391	0.00074232	0.00909337
YPL257W	YPL257W	-0.5691391	5.5928115	0.004535	0.03429492

YBL072C	RPS8A	-0.5676218	5.49855876	0.00342751	0.02779949
YGL187C	COX4	-0.5613429	5.04440815	0.00587832	0.04055659
YBL066C	SEF1	-0.5495324	6.9393763	0.00467163	0.03471052
YEL064C	AVT2	-0.5450972	5.20509736	0.00285236	0.02405265
YHR006W	STP2	-0.5257087	5.6682942	0.00513632	0.03687391
YBL054W	TOD6	-0.5208489	5.96832296	0.00617653	0.04193334
YPL188W	POS5	0.4770298	6.14766835	0.00550033	0.0386374
YCR035C	RRP43	0.4910092	6.00807669	0.00378398	0.02981314
YKL133C	RCI50	0.49110415	6.22000899	0.00406115	0.03126793
YKL171W	NNK1	0.49570789	8.77221756	0.00647106	0.04351585
YIL091C	UTP25	0.49577281	6.65443661	0.00461974	0.03468889
YKL116C	PRR1	0.49669378	5.40732839	0.00659191	0.04418866
YIL074C	SER33	0.50685781	5.36595997	0.00638857	0.04323478
YML027W	YOX1	0.5077152	5.14991918	0.00700788	0.04653672
YKL126W	YPK1	0.51288743	5.35043746	0.00595907	0.04098064
YGL233W	SEC15	0.514023	8.18015305	0.00580595	0.04018776
YPL180W	TCO89	0.52021632	5.32671807	0.00501012	0.03647416
YBL086C	YBL086C	0.53331119	8.62505944	0.0049316	0.03613671
YEL063C	CAN1	0.55173389	7.84900842	0.00713252	0.04707023
YNL323W	LEM3	0.56698516	7.45825371	0.00689292	0.04591681
YAR028W	YAR028W	0.56914621	5.97823795	0.00088477	0.01002352
YIL123W	SIM1	0.5698579	8.10187758	0.00163104	0.0156831
YNL327W	EGT2	0.57292801	6.2586786	0.00395754	0.03073247
YHR061C	GIC1	0.57644706	4.94617425	0.00303625	0.02510516
YOL105C	WSC3	0.57769491	6.61611571	0.00601666	0.04111065
YBL074C	AAR2	0.57803819	7.77417004	0.00236704	0.02087121
YML123C	PHO84	0.57892632	6.17370401	0.00356333	0.02857387
YJL216C	IMA5	0.58522529	5.8983673	0.00501198	0.03647416
YBL017C	PEP1	0.58838075	8.11927857	0.00464867	0.03471052
YEL022W	GEA2	0.58982382	8.35452261	0.00391549	0.03058975
YGL250W	RMR1	0.60302727	6.74778649	0.00482792	0.03562267
YLR019W	PSR2	0.60333389	5.30868985	0.00662623	0.04427906
YAL046C	BOL3	0.60602151	5.70373111	0.00094526	0.01046182
YEL039C	CYC7	0.60808248	3.87812258	0.00752947	0.04923114
YOL139C	CDC33	0.61202356	5.79340305	0.0042855	0.03264044
YBL084C	CDC27	0.61360499	10.1510117	0.00512862	0.03687391
YOL135C	MED7	0.61486549	6.47714433	0.00238198	0.02091613
YJL118W	YJL118W	0.61656743	5.5633903	0.00071907	0.00893585
YLL010C	PSR1	0.61800856	7.7618937	0.00077477	0.00930157
YDL194W	SNF3	0.62120893	7.80044702	0.00175918	0.0166887
YDL216C	RRI1	0.6225493	8.24333818	0.00113472	0.01186645
YLR024C	UBR2	0.62721682	8.37498647	0.00093118	0.01036

YFR032C	RRT5	0.62759434	4.33197737	0.00531848	0.03767259
YJL155C	FBP26	0.64753646	7.82356324	0.00042691	0.00608852
YGL253W	HXK2	0.64862276	6.07242664	0.00053368	0.00722339
YNL278W	CAF120	0.65811801	7.8157986	0.00185416	0.01735724
YEL013W	VAC8	0.66970468	8.05471469	0.00022719	0.00357608
YGL167C	PMR1	0.67001783	7.7366025	0.00054877	0.00738065
YJL162C	JJJ2	0.67321312	6.75266954	0.00088881	0.01002352
YCR008W	SAT4	0.67500752	8.24685481	0.00020171	0.00327196
YAL013W	DEP1	0.68260202	7.71540993	0.00020729	0.00333707
YBL102W	SFT2	0.68277947	4.73979066	0.00316278	0.02601816
YLL035W	GRC3	0.68432392	9.03536405	0.00059044	0.0076974
YDL222C	FMP45	0.68589124	6.94647916	0.00038408	0.00562879
YIL083C	CAB2	0.68636261	3.43923957	0.00528463	0.03767094
YNL304W	YPT11	0.68888264	6.85258081	0.0005745	0.00754783
YPL263C	KEL3	0.68934881	8.7008953	0.00317115	0.02601816
YKL198C	PTK1	0.70009334	9.02400845	0.0005036	0.00690424
YPL255W	BBP1	0.71128056	7.82676584	0.00073853	0.00909337
YHL019C	APM2	0.71129052	9.10729239	0.00047606	0.00656905
YCL029C	BIK1	0.72352535	8.94847464	0.0003428	0.00509406
YCR044C	PER1	0.72411789	4.4513562	0.00241948	0.02115801
YNL229C	URE2	0.72710368	3.57539834	0.00265052	0.02289577
YML102W	CAC2	0.73011932	8.94984482	0.00029416	0.00446496
YJL121C	RPE1	0.73048393	4.8393722	0.00350287	0.02830266
YAL039C	CYC3	0.73888367	8.6908152	0.0002558	0.00393891
YNL243W	SLA2	0.75471881	7.6841601	0.00010057	0.00176783
YAL044W-A	BOL1	0.75589093	6.21660425	3.21E-05	0.00064278
YGL228W	SHE10	0.76093316	9.27021493	0.0001405	0.00233248
YLL016W	SDC25	0.76148467	8.03728629	0.00035783	0.00528043
YFL011W	HXT10	0.77586756	7.28042776	0.00016277	0.00268126
YDL148C	NOP14	0.77641164	7.56050496	4.19E-05	0.00081637
YML076C	WAR1	0.79526706	7.81697342	8.32E-05	0.00152413
YFR025C	HIS2	0.81447213	3.13994237	0.00279029	0.0237175
YER034W	YER034W	0.81671127	4.22321979	0.00010542	0.00182127
YAL055W	PEX22	0.82389071	6.24649624	1.37E-05	0.00030985
YPL204W	HRR25	0.83268138	11.8544915	0.00736144	0.0484305
YOL136C	PFK27	0.83987089	8.4311123	8.06E-06	0.00020161
YEL046C	GLY1	0.85328791	8.13912609	7.67E-06	0.00019642
YBL057C	PTH2	0.85725558	6.25454295	8.14E-05	0.00150484
YOL083W	ATG34	0.85979695	9.56405517	0.00047201	0.00655574
YCR026C	NPP1	0.86079361	12.1913253	0.00530052	0.03767094
YOL075C	YOL075C	0.86479174	7.72786837	2.60E-06	7.57E-05
YGL203C	KEX1	0.87355633	7.63014841	1.36E-05	0.00030979

YBL056W	PTC3	0.88814983	6.49979774	1.06E-05	0.00024973
YNL256W	FOL1	0.89403316	11.965568	0.00378802	0.02981314
YAR010C	YAR010C	0.90525032	11.8828381	0.00372433	0.02953062
YER007W	PAC2	0.90774583	11.9730482	0.00355305	0.02857387
YKL134C	OCT1	0.91370153	7.06262368	1.39E-05	0.00031118
YHR028C	DAP2	0.91400117	11.7210782	0.00234282	0.0207868
YDL164C	CDC9	0.92252131	11.7051508	0.00215277	0.0194665
YDL229W	SSB1	0.9439178	8.51429037	0.00298408	0.02477021
YPL189W	GUP2	0.95186584	6.90660997	2.81E-06	8.06E-05
YFR010W	UBP6	0.9531402	11.6215219	0.00160377	0.01549099
YML113W	DAT1	0.95521956	7.88790069	0.00562962	0.03909458
YKL151C	NNR2	0.9600562	11.6830084	0.00187308	0.01745742
YKL178C	STE3	0.97130262	7.00015205	9.60E-07	3.00E-05
YIL092W	YIL092W	0.98046922	6.95706131	1.57E-06	4.71E-05
YHR005C	GPA1	0.98696352	6.82336922	3.12E-07	1.09E-05
YCL001W-B	YCL001W-B	0.99762503	4.48649061	8.04E-05	0.00149791
YCL001W-A	YCL001W-A	1.01370204	5.50229303	1.74E-05	0.00037647
YBL093C	ROX3	1.02932211	7.9106926	0.00254823	0.02210198
YOL149W	DCP1	1.04659213	5.05548575	5.45E-05	0.00103328
YML054C	CYB2	1.04718401	10.9189563	0.000212	0.00338728
YER017C	AFG3	1.06485822	7.03335287	5.08E-07	1.69E-05
YAR035W	YAT1	1.07988474	7.0870982	1.19E-07	4.37E-06
YAL020C	ATS1	1.0809253	6.52896115	5.42E-08	2.05E-06
YFL044C	OTU1	1.09014622	7.15283875	0.00083983	0.00975218
YGL242C	YGL242C	1.12479315	7.12255672	0.00070398	0.00885177
YNL314W	DAL82	1.12987554	7.86881234	0.00096418	0.01061595
YLR008C	PAM18	1.13548911	10.6366373	0.00010198	0.00177631
YML043C	RRN11	1.20016284	6.36182902	3.75E-08	1.47E-06
YFL068W	YFL068W	1.44107905	2.26136647	0.0001189	0.00200534

Supplementary table 5. Metabolites determined by NMR.

	Concentrations (µM)															
Compound*	1	2	3	4	5	6	7	8	9	10	11	12	13	14	15	16
Compound ID	284	1713	1714	1716	9	272	240	22	101423	117	205	32	2410	286	258	229
A1	0.8	4.8	3.6	10	7.9	2.4	11.5	7.4	3.1	2.7	6.3	28	0.9	2.8	55.6	5.7
A2	0.9	3.6	2.9	6.3	15.5	2.1	4.5	6.7	3.4	2.1	5.1	33.2	0.6	1	15.1	7.5
A3	0.8	4.6	8	8.2	26.9	1.7	10.6	8	5	4.2	8.6	42.6	2.2	3.6	21	6
E1	0.7	6.4	5.9	11.4	11.8	4.6	14.5	7.4	3.3	5	11	30.6	2	3.6	38.6	9.5
E2	0.8	5.6	5.3	6.9	15	4.2	15.2	2.9	4	1.9	7.4	34.3	1.1	4.2	52.6	10
E3	0.9	8.2	7.6	9.1	9.3	3.7	15	4.5	6.2	3.3	13.3	25.6	1.4	5.6	123	16.5
G1	0.7	6.7	5.3	5.5	11.1	4.3	12.6	3.3	5.9	5.3	8.4	29.9	0.8	4.8	5.9	13.8
G2	0.7	8.2	5.9	14.6	14.5	5.7	11	8.5	9.4	7.5	6.4	32.8	1.7	4.4	15.6	15.6
G3	0.9	6.2	7.7	6.2	31	3.6	14.7	2.9	4.2	5.9	9.9	41.1	2.5	3.6	7.9	11.6
AG1	0.7	8.9	6.6	11.7	11.1	2.1	7.6	9.4	4.8	7.8	11.3	30.6	1.7	3.4	13.5	7.2
AG2	0.7	6.5	7.6	11.9	10.1	1.6	13	8.1	5.7	7.6	9.5	29.7	2.2	3.4	6.4	12.4
AG3	0.9	6.3	13.7	9.6	26.9	1	15.9	12	6.5	8.2	9.6	44.3	2.9	2.6	11.8	10.8

*Compound: 1- 3-Hydroxyisovalerate; 2- ADP; 3- AMP; 4- ATP; 5- Acetate; 6- Adenine; 7- Arginine; 8- Citrate; 9- Coenzyme A;10- Ornithine;11- Ethanol;12- Formate;13- GTP;14- Galactitol;15- Galactose; 16- Glutamate;17- Glycerol; 18- Histidine;19- Lactate;20- Lysine;21- NAD+;22- Nicotinurate;23- O-Phosphocholine;24- Succinate;25- Threonine;26- Trehalose;27- UDP-N-Acetylglucosamine;28- UDP-galactose;29- UDP-glucose;30- UMP;31- Valine; 32- sn-Glycero-3-phosphocholine. Compound ID: Control cells with empty vector– E, Control cells with empty vector + 10 µM genipin – EG, αSyn-GFP cells – A, αSyn-GFP cells + 10 µM genipin – AG.

	Concentrations (µM)															
Compound*	17	18	19	20	21	22	23	24	25	26	27	28	29	30	31	32
Compound ID	36	217	42	291	499	2781	321	60	219	1776	4553	1255	1287	3341	215	333
A1	4.6	1.9	3.3	17.5	0.7	5.3	0.8	1.4	2.6	5.7	4.9	2.4	5	1.5	0.8	0.6
A2	3.9	1.2	2	14.4	0.5	3.4	0.5	1	1.2	5.8	4.3	2.8	4.8	4.5	0.3	1.3
A3	3.4	0.9	2.8	25.7	0.8	6.3	0.7	1.4	2.5	6.5	2.8	1.6	4	5	0.6	0.5
E1	4.9	5.6	4.1	33.1	0.7	8.1	1	1.7	5.7	4.6	1.3	3.4	7	4	1	0.7
E2	9.3	4.4	4.3	16.6	0.7	6.9	0.9	1.6	3.5	2.5	3.2	3.9	7.5	3.6	0.9	0.7
E3	8.5	7.3	3.1	25	1.1	8.9	0.9	2.8	5.1	5.9	3.6	3	7.2	5.1	1.6	0.9

G1	5.6	5.5	3.3	36.2	1	6.9	0.8	1.7	3.2	3.7	5.3	2.6	6.9	3.4	0.6	0.5
G2	4.6	8.2	3	50.7	1.5	11	1	1.6	3.6	1.3	4	5.3	8.5	6.1	0.6	0.6
G3	3.6	4.9	2	41.7	-	3.8	0.8	1.7	2.8	2.7	2.1	1.3	2.4	6.7	0.7	0.4
AG1	5.2	2	5.2	24.4	-	7.2	0.4	1.4	2.9	0.7	4.8	2.6	5.8	3.6	-	0.5
AG2	4.9	2.2	3.4	40.1	1.5	8.9	0.7	1.6	3.8	6.8	6.1	3.8	5.5	2.5	-	0.4
AG3	4.1	2.4	4	43	1.2	12.5	1	1.9	2.8	6.3	5.7	1.8	4.7	7.6	0.7	0.6

*Compound: 1- 3-Hydroxyisovalerate; 2- ADP; 3- AMP; 4- ATP; 5- Acetate; 6- Adenine; 7- Arginine; 8- Citrate; 9- Coenzyme A;10- Ornithine;11- Ethanol;12- Formate;13- GTP;14- Galactitol;15- Galactose; 16- Glutamate;17- Glycerol; 18- Histidine;19- Lactate;20- Lysine;21- NAD+;22- Nicotinurate;23- O-Phosphocholine;24- Succinate;25- Threonine;26- Trehalose;27- UDP-N-Acetylglucosamine;28- UDP-galactose;29- UDP-glucose;30- UMP;31- Valine; 32- sn-Glycero-3-phosphocholine. Compound ID: Control cells with empty vector– E, Control cells with empty vector + 10 μM genipin – EG, αSyn-GFP cells – A, αSyn-GFP cells + 10 μM genipin – AG.

Supplementary table 6. List of reagents.

Item Name	Brand	Catalog #
Reagents		
Acetone, 99.6%, ACS reagent	Acros Organics	423240025
Acetonitrile, HPLC for gradient analysis	Fisher Chemical	A/0627/17
Acrylamide, suitable for electrophoresis, >=99%	Sigma-Aldrich	A8887-500G
Agarose	Fisher BioReagents	BP160-500
CSM (powder)	MP Biomedicals	4500022
CSM-Ura (powder)	MP Biomedicals	4511212
D-(+)-Galactose, suitable for microbiology, >=99.0%	Merck Millipore	48260-500G-F
D-(+)-Glucose, powder, BioReagent	Sigma-Aldrich	G7021-1KG
D-(+)-Raffinose pentahydrate, suitable for microbiology, >=99.0%	Sigma-Aldrich	83400-100G
Yeast Nitrogen Base without Amino Acids	BD	291920
Dimethyl sulfoxide (DMSO)	Merck	1.02952.1000
Ethanol absolute for analysis	Merk	1.00983.2511
Glass beads, acid-washed, 425-600 mum (30-40 U.S. sieve)	Sigma-Aldrich	G8772-100g
Glycine, 1 kg	Carl Roth	3908.2
Methanol	Carlo Erba	414816

SDS (Dodecyl sulfate sodium salt)	Sigma-Aldrich	8220501000
Thioflavin T	Sigma	T3516
Trichloroacetic acid (TCA)	Sigma-Aldrich	T6399-250g
TRITON X-100 for analysis	Merck	1.08603.1000
Bovine Serum Albumin, heat shock fraction, pH 7, >=98%	Sigma-Aldrich	A7906-100G
Gallic acid	Sigma-Aldrich	G7384
Genipin	Carbosynth	FG30976
cOmplete Tablets, Mini EDTA-free EASYpack - Protease Inhibitor Cocktail Tablets	Roche	4693159001
PhosSTOP EASYpack	Roche	4906837001
Protein Marker VI	Panreac Applichem	P100502
FM™ 4-64 Dye	Invitrogen	T13320
CellView Blue CMAC	Tebu-bio	C037
Dihydroethidium (DHE)	Life technologies	D11347
Alfa-synuclein	rPEPTIDE	S-1001-2
Antibodies		
Purified Mouse Anti-α-Synuclein Clone 42/α-Synuclein (RUO)	BD Pharmingen	610787
PGK1 monoclonal antibody	Invitrogen	459250
GAPDH Loading control Antibody (GA1R)	Invitrogen	MA5-15738
Carboxypeptidase Y Monoclonal Antibody (anti-CPY)	Invitrogen	A-6428
Anti-GFP (anti-GFP (3H9)	Chromotek	3H9
Anti-alpha-tubulin (AA4.3)	Developmental Studies	AB_579793
