# Supplementary material for: Genipin prevents alpha-synuclein aggregation and toxicity by affecting endocytosis, metabolism and lipid storage
Source: Nat Commun. 2023 Apr 6;14:1918. doi: 10.1038/s41467-023-37561-2 (PMC10079842; doi:10.1038/s41467-023-37561-2)
Supplement: Supplementary file 3 — Reporting Summary [file 41467_2023_37561_MOESM3_ESM.pdf]

nature portfolio

Corresponding author(s): Cláudia Nunes dos Santos

Last updated by author(s): Mar 16, 2023

Reporting Summary

Nature Portfolio wishes to improve the reproducibility of the work that we publish. This form provides structure for consistency and transparency in reporting. For further information on Nature Portfolio policies, see our Editorial Policies and the Editorial Policy Checklist.

Statistics

For all statistical analyses, confirm that the following items are present in the figure legend, table legend, main text, or Methods section.

n/a	Confirmed	
☐	☒	The exact sample size (*n*) for each experimental group/condition, given as a discrete number and unit of measurement
☐	☒	A statement on whether measurements were taken from distinct samples or whether the same sample was measured repeatedly
☐	☒	The statistical test(s) used AND whether they are one- or two-sided<br>*Only common tests should be described solely by name; describe more complex techniques in the Methods section.*
☒	☐	A description of all covariates tested
☒	☐	A description of any assumptions or corrections, such as tests of normality and adjustment for multiple comparisons
☐	☒	A full description of the statistical parameters including central tendency (e.g. means) or other basic estimates (e.g. regression coefficient) AND variation (e.g. standard deviation) or associated estimates of uncertainty (e.g. confidence intervals)
☐	☒	For null hypothesis testing, the test statistic (e.g. *F*, *t*, *r*) with confidence intervals, effect sizes, degrees of freedom and *P* value noted<br>*Give P values as exact values whenever suitable.*
☒	☐	For Bayesian analysis, information on the choice of priors and Markov chain Monte Carlo settings
☒	☐	For hierarchical and complex designs, identification of the appropriate level for tests and full reporting of outcomes
☒	☐	Estimates of effect sizes (e.g. Cohen's *d*, Pearson's *r*), indicating how they were calculated

Our web collection on statistics for biologists contains articles on many of the points above.

Software and code

Policy information about availability of computer code

Data collection	Confocal microscopy – ZEN black version 2011<br>Flow Cytometry - Cube (Partec) version 6<br>Yeast Optical Density – Gen5 version 2.0<br>Western Blot – ImageStudio version 5.2.5
Data analysis	Data treatment and organization – Microsoft Excel version 16.69.1<br>Image treatment and cell counting – ImageJ, Cell Counting Plug in version 2.3.0/1.53q<br>Data treatment, statistics and graphs – GraphPad version 6<br>Flow Cytometry – FlowJo 10<br>NMR – Metabolite identification and quantification were performed recurring to ChenomxNMRsuite8.12.<br>RNAseq – FastQC, Bowtie2 and SAMtools v.0.1.19 packages were used.<br>Yeast Growth Curves – R and R Studio version 0.99.902. Plugin described in doi:10.3390/antiox9090789<br>Fly walker – FlyWalker software package described in doi: 10.7554/eLife.00231<br>Control and process data from Thermo Scientific™ LC-MS systems-Xcalibur software package 2.0

For manuscripts utilizing custom algorithms or software that are central to the research but not yet described in published literature, software must be made available to editors and reviewers. We strongly encourage code deposition in a community repository (e.g. GitHub). See the Nature Portfolio guidelines for submitting code & software for further information.

Data

Policy information about availability of data

All manuscripts must include a data availability statement. This statement should provide the following information, where applicable:

- Accession codes, unique identifiers, or web links for publicly available datasets
- A description of any restrictions on data availability
- For clinical datasets or third party data, please ensure that the statement adheres to our policy

The RNAseq data generated and used was deposited and publicly available under accession code GSE226744 [https://www.ncbi.nlm.nih.gov/geo/query/acc.cgi?acc=GSE226744]. The NMR data of yeast metabolites is available at https://github.com/lgafeira/Genipin-alphaSynuclein. The LC-MS data of genipin was deposited in MassIVE under the accession code MSV000091485 (doi: 10.25345/C5NS0M75B). The reporting summary for this article is available in the Supplementary Information section. All the other data supporting this study are available within this Article, Supplementary Information, and Source Data file. Source data are provided with this paper. Yeast Growth Curves – R and R Studio plugin is fully described in doi:10.3390/antiox9090789 (https://www.mdpi.com/2076-3921/9/9/789). FlyWalker software package described in doi:10.7554/eLife.00231 (https://elifesciences.org/articles/00231).

Field-specific reporting

Please select the one below that is the best fit for your research. If you are not sure, read the appropriate sections before making your selection.

☒ Life sciences ☐ Behavioural & social sciences ☐ Ecological, evolutionary & environmental sciences

For a reference copy of the document with all sections, see nature.com/documents/nr-reporting-summary-flat.pdf

Life sciences study design

All studies must disclose on these points even when the disclosure is negative.

Sample size	No sample-size calculation was performed in this study. The sample size used for each study was based on the sample size described in the literature for the type of assay regarding the number of cells counted (https://doi.org/10.1371/journal.pgen.1004302) and the number of flies used (Doi:10.7554/eLife.00231 and doi: 10.1093/hmg/ddu606).
Data exclusions	For Fly walker experiments using Drosophila melanogaster, outliers are defined as any value that is 1.5 times the interquartile range below or above the 25% and 75% quartiles, respectively. Statistical analysis was performed using custom Python scripts and GraphPad Prism.
Replication	The replication of the results was verified by performing as least three independent biological experiments.
Randomization	For biochemical or cell-based experiments no randomization of samples were required. Only samples of leaves of C. album L. collecting was done randomly
Blinding	Blinding was apllied during data analysis. In cell counting the files' names were randomized prior to cell counting by a person not involved in the counting. Files' names were given upon cell counting.

Reporting for specific materials, systems and methods

We require information from authors about some types of materials, experimental systems and methods used in many studies. Here, indicate whether each material, system or method listed is relevant to your study. If you are not sure if a list item applies to your research, read the appropriate section before selecting a response.

Materials & experimental systems

n/a	Involved in the study	
☐	☒	Antibodies
☒	☐	Eukaryotic cell lines
☒	☐	Palaeontology and archaeology
☐	☒	Animals and other organisms
☒	☐	Human research participants
☒	☐	Clinical data
☒	☐	Dual use research of concern

Methods

n/a	Involved in the study	
☒	☐	ChIP-seq
☐	☒	Flow cytometry
☒	☐	MRI-based neuroimaging

Antibodies

Antibodies used	The following antibodies were used in this study:<br>- Anti-alpha synuclein (Purified Mouse Anti-α-Synuclein, Clone 42/α-Synuclein (RUO), BD Biosciences, Catalog Number 610787);<br>- Anti-Pgk1 (Invitrogen, Clone 22C5D8, Catalog number 459250, Lot UD2749418);

- Anti-GAPDH (GAPDH Loading control Antibody, Clone GA1R, Thermo Fisher Scientific, Catalog number MA5-15738, Lot TH270579);
- Anti-CPY (Carboxypeptidase Y Monoclonal Antibody, Clone 10A5B5, Invitrogen, Catalog number A-6428, Lot 2286908);
- Anti-GFP (anti-GFP (3H9), Chromotek, Catalog number: 3H9)
- Anti-alpha-tubulin (AA4.3, Developmental Studies Hybridoma Bank)

For further information, please check Methods section of the manuscript.

Validation

The following information is provided by the indicated suppliers regarding the antibodies used in this study:

- Anti-alpha synuclein - The 140 amino acid protein (α-Synuclein) is the human homologue of rat Synuclein-1 and is identical to the non-amyloid-β component precursor (NACP), a presynaptic protein involved in amyloidogenesis in Alzheimer's disease (AD). Although the exact function of the Synucleins has not been determined, they have been linked to the prominent neurodegenerative disorders AD and Parkinson's disease. This antibody is routinely tested by western blot analysis.

- Anti-Pgk1 – Also known as ATP:3-phosphoglycerate 1-phosphotransferase showed that the plasmin reductase isolated from conditioned medium of fibrosarcoma cells is the glycolytic enzyme phosphoglycerate kinase. They concluded that phosphoglycerate kinase not only functions in glycolysis but is secreted by tumor cells and participates in the angiogenic process as a disulfide reductase. This product reacts with Saccharomyces cerevisiae PGK1 - predicted molecular weight: 45 kDa. Purity >95% by SDS-PAGE.

- Anti-GAPDH – This antibody detects GAPDH from BL-21 bacteria, Sf9 insect, Saccharomyces cerevisiae (yeast), human, mouse, rat, rabbit, hamster, and chicken samples. MA5-15738 has been successfully used in Western blot, ICC, IF, IHC (P), FACS and ELISA.

- Anti-CPY – Antibody binding specificity was determined by Particle Concentration Fluorescence Immunoassay (PCFIA) using the yeast caboxypeptidase, by Western blot immunoassay using protein extracts from yeast, and by indirect immunofluorescence of fixed yeast cells. The antibody can be used to detect the yeast carboxypeptidase on Western blots or to immunolocalize the carboxypeptidase in fixed yeast cells. The antibody is ideal for screening for caroboxypeptidase secretion (Vps-phenotype) in yeast using the colony immunoblot overlay assay. The antibody has been extensively used in screens for new vps mutations, as well as in complementation analysis with the vps mutant collection.

- Anti-GFP – Rat monoclonal antibody [3H9] to Green Fluorescent Proteins. The antibody recognizes green fluorescent proteins derived from Aequorea victoria GFP, such as eGFP, wtGFP, YFP, or CFP.

- Anti-alpha-tubulin – Initial characterization [PMID 6363422] performed on Naegleria gruberi tubulins suggested that this antibody target was beta-tubulin. Further characterization [PMID 3654753] demonstrated that the target was alpha-tubulin.

Animals and other organisms

Policy information about studies involving animals; ARRIVE guidelines recommended for reporting animal research

Laboratory animals	Drosophila melanogaster, w1118 strain was used as background, sex and age described in methods section
Wild animals	No wild animals were used in this study.
Field-collected samples	Leaves of C. album L. were collected by random sampling in an extensive area of Comporta (southern region of Portugal).
Ethics oversight	This study did not required ethicals approval.

Note that full information on the approval of the study protocol must also be provided in the manuscript.

Flow Cytometry

Plots

Confirm that:

☒ The axis labels state the marker and fluorochrome used (e.g. CD4-FITC).

☒ The axis scales are clearly visible. Include numbers along axes only for bottom left plot of group (a 'group' is an analysis of identical markers).

☒ All plots are contour plots with outliers or pseudocolor plots.

☒ A numerical value for number of cells or percentage (with statistics) is provided.

Methodology

Sample preparation	For the determination of cell death, yeast cells were incubated with 5 µg.mL−1 of propidium iodide, for 30 min protected from light, under agitation. For superoxide quantification, cells were incubated with 30 µM of Dihydroethidium, for 15 min at 30°C, with agitation and protected from light. Results of cell viability and superoxide levels were expressed as frequency of positive cells and median fluorescence intensity, respectively.
Instrument	The cytomer used was Partec Cube6.
Software	The software used was Cube6 for acquisition and FlowJo for data treatment.

Cell population abundance	Data analysis was performed using FlowJo software and a minimum of 100,000 events were collected for each experiment.
Gating strategy	Gating strategy was based on both negative and positive controls for both of stainings used.

☒ Tick this box to confirm that a figure exemplifying the gating strategy is provided in the Supplementary Information.
